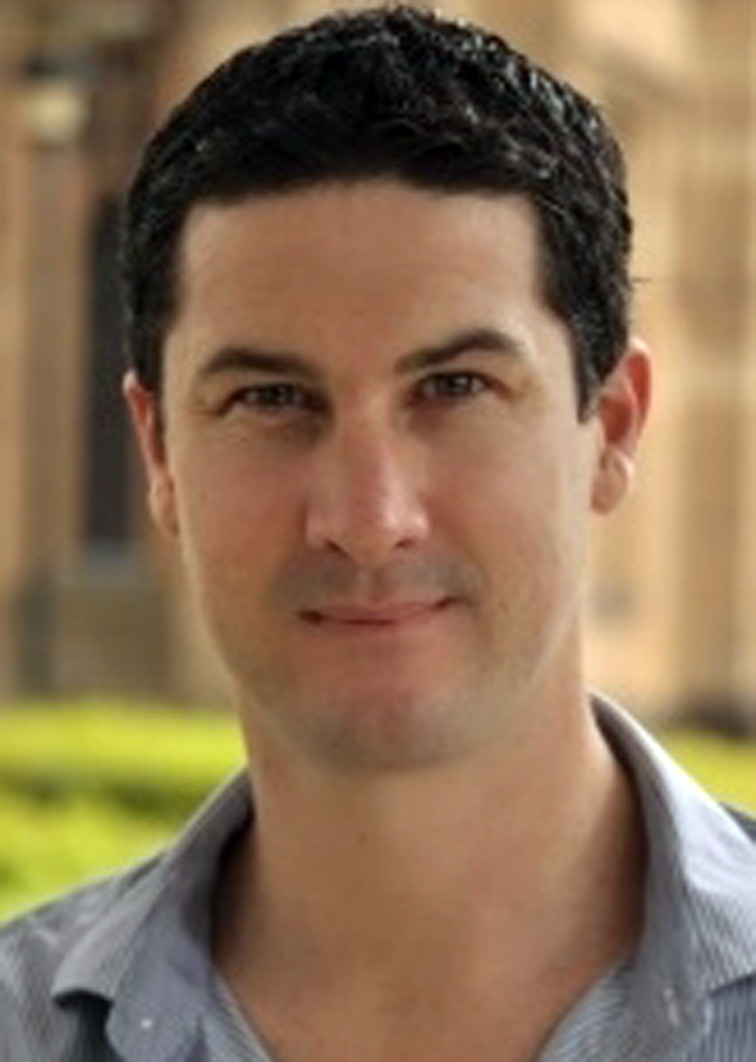# Chemische Synthese und Semisynthese von lipidierten Proteinen

**DOI:** 10.1002/ange.202111266

**Published:** 2022-02-03

**Authors:** Cameron C. Hanna, Julia Kriegesmann, Luke J. Dowman, Christian F. W. Becker, Richard J. Payne

**Affiliations:** ^1^ School of Chemistry The University of Sydney Sydney NSW 2006 Australien; ^2^ Institut für Biologische Chemie Fakultät für Chemie Universität Wien Wien Österreich; ^3^ Australian Research Council Centre of Excellence for Innovations in Peptide and Protein Science The University of Sydney Sydney NSW 2006 Australien

**Keywords:** chemische Proteinsynthese, lipidiertes Protein, Lipopeptide, Posttranslationale Modifikation, Semisynthese

## Abstract

Lipidierung ist eine ubiquitäre Modifikation von Peptiden und Proteinen, die entweder co‐ oder posttranslational auftreten kann. Für die Vielzahl von Lipidklassen wurde gezeigt, dass diese viele entscheidende biologische Aktivitäten, z. B. die Regulierung der Signalweiterleitung, Zell‐Zell‐Adhäsion sowie die Anlagerung von Proteinen an Lipid‐Rafts und Phospholipidmembranen, beeinflussen. Während die Natur Enzyme nutzt, um Lipidmodifikationen in Proteine einzubringen, ist ihre Nutzung für die chemoenzymatische Herstellung von lipidierten Proteinen häufig ineffizient. Eine Alternative ist die Kombination moderner synthetischer und semisynthetischer Techniken, um lipidierte Proteine in reiner und homogen modifizierter Form zu erhalten. Dieser Aufsatz erörtert Fortschritte in der Entwicklung der Lipidierungs‐ und Ligationschemie und deren Anwendung in der Synthese und Semisynthese homogen lipidierter Proteine, die es ermöglichen, den Einfluss dieser Modifikationen auf die Proteinstruktur und ‐funktion zu untersuchen.

## Graphical abstract

Lipidierung ist eine co‐ und posttranslationale Modifikation, die Struktur, Funktion und Lokalisierung von Peptiden und Proteinen beeinflusst. Dieser Aufsatz diskutiert die wichtigsten Fortschritte bei der Entwicklung moderner synthetischer und semisynthetischer Methoden zur Herstellung homogen lipidierter Proteine, die die Untersuchung der Auswirkungen von Lipidmodifikationen auf ihre Struktur und Funktion mit beispielloser Präzision ermöglicht haben.

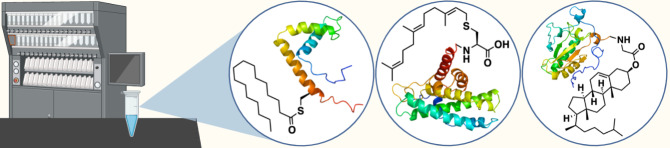

## 1 Einleitung

Proteine sind für die Organisation der meisten biologischen Prozesse in lebenden Systemen verantwortlich. Fortschritte in Genetik und Proteomik haben gezeigt, dass die Gesamtzahl der Proteine einer Zelle (das Proteom) weit größer ist als die Anzahl genetisch codierter Proteine. Beispielsweise codieren im Menschen ungefähr 20 000 Gene über eine Million Proteinprodukte.[^1^, ^2^, ^3^] Diese enorme Diversifikation von genetischen Informationen ist größtenteils das Ergebnis von co‐ oder posttranslationalen Modifikationen (PTMs), die entweder während oder nach der Proteinsynthese am Ribosom auftreten.[^1^, ^3^] Hunderte verschiedene PTMs sind bis heute entdeckt worden, und sie können durch enzymatische oder nicht‐enzymatische Prozesse auftreten.[^4^, ^5^] Die Natur dieser Modifikationen variiert von der Anbringung kleiner Gruppen (z. B. Phosphorylierung, Methylierung, Sulfatierung oder Acetylierung) bis zur Anbringung von größeren und/oder strukturell komplexen Biomolekülen (z. B. Ubiquitylierung, Glykosylierung, ADP‐Ribosylierung oder Lipidierung).[^1^, ^6^] Andere häufige PTMs beinhalten kleine Modifikationen von Aminosäureseitenketten (z. B. Citrullinierung), Polypeptid‐Spaltung oder Zyklisierungen.^[5]^ Während es immer mehr Beweise gibt, dass PTMs an einem Großteil aller humanen Proteine zu finden^[7]^ und diese notwendig für Struktur, Lokalisierung und/oder biologische Funktion sind (einschließlich der Wirksamkeit vieler Biologika),^[8]^ bleiben die modulierenden Wirkungen von PTMs für den Großteil des Proteoms unbekannt.[^2^, ^3^]

Lipidierung ist eine weitverbreitete Modifikation von Proteinen, die entweder co‐ oder posttranslational auftreten kann. Charakteristisch für diese Modifikation ist die Addition von Kohlenwasserstoffketten verschiedener Längen, sodass die Hydrophobie der Proteine erhöht wird, was häufig zur Verankerung in Membranen führt.[^9^, ^10^] Diese Lokalisierung an Zell‐ oder intrazellulären Membranen besitzt verschiedene Funktionen, wie zum Beispiel die Modulierung der Aktivität von Signalproteinen, die räumliche Trennung eines Proteins von einem Substrat oder die Verstärkung der Protein‐Substrat‐Assoziation durch Membran‐Clusterbildung.[^9^, ^10^] Zusätzlich zu den weitreichenden Rollen von Protein‐gebundenen Lipiden im Bereich der Biologie werden diese Moleküle auch mit Krankheiten in Verbindung gebracht. Beispielsweise ist die Pathogenität des humanen Onkoproteins Src darauf zurückzuführen, dass die Lipidierung einen Transport des Proteins zur Plasmamembran auslöst.^[11]^

**Figure 1:**
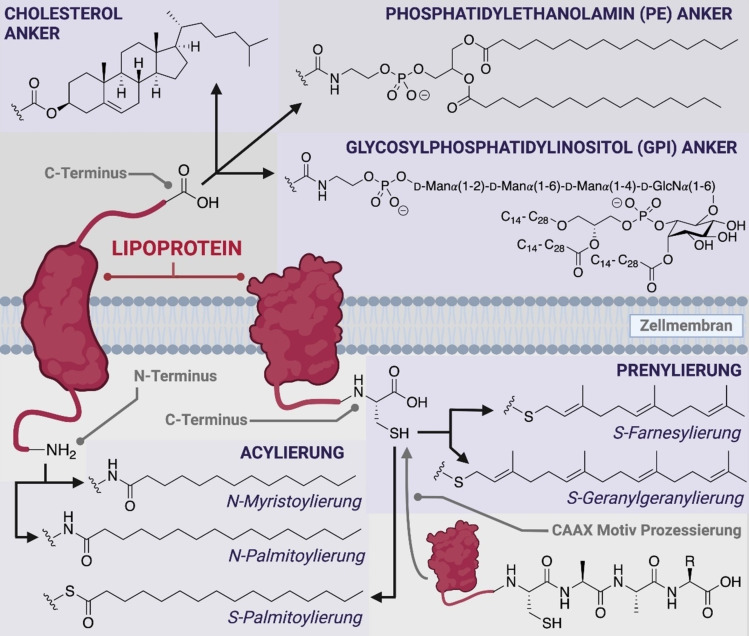
Überblick über die verschiedenen Klassen von co‐ und posttranslationalen Peptid‐ und Protein‐Lipidmodifikationen. Prenylierung kann zusätzlich zu integralen Membranproteinen auch bei cytosolischen Proteinen auftreten (nicht gezeigt).

Lipidmodifikationen umfassen Prenylierung an Cystein (Cys)‐Seitenketten (z. B. *S*‐Farnesyl‐ und *S*‐Geranylgeranyl‐Lipide), Fettsäure‐Acylierung an Cysteinseitenketten oder dem N‐Terminus (z. B. *S*‐ und *N*‐Palmitoyl‐ oder *N*‐Myristoyl‐Lipide) sowie das Anbringen von Cholesterol‐, Glykosylphosphatidylinositol (GPI)‐ oder Phosphatidylethanolamin (PE)‐Ankern an den C‐Terminus (Abbildung 1). Angesichts der funktionellen Bedeutung von Proteinlipidierung und der Diversität an Lipidmodifikationen, die in der Natur existieren, werden Strategien benötigt, die den Zugang zu diesen Biomolekülen in homogener Form erleichtern und somit detaillierte Strukturfunktionsstudien ermöglichen.^[12]^ Dieser Übersichtsartikel soll die biologische Bedeutung der Proteinlipidierung hervorheben und einen detaillierten Überblick über die synthetischen und semisynthetischen Methoden geben, die entwickelt und eingesetzt wurden, um einen effizienten Zugang zu dieser Klasse von modifizierten Proteinen zu erhalten.

### 1.1 Prenylierung

Prenylierung ist charakterisiert durch die Anbringung von mehreren Isopreneinheiten an Cysteinseitenketten in der C‐terminalen Region von Proteinen über eine Thioetherbindung. Die beiden Arten der Prenylierung sind Farnesylierung und Geranylgeranylierung, welche drei bzw. vier Isopren‐Einheiten beinhalten (Abbildung 1). In Säugetierzellen sind bis zu 2 % aller zellulären Proteine prenyliert und die meisten davon sind geranylgeranyliert.^[13]^ Prenyl‐Modifikationen rekrutieren ansonsten lösliche Proteine an Zellmembranen. Hierbei kann es sich entweder um die Plasmamembran oder um Endomembranen handeln, die Organellen wie Golgi, endoplasmatisches Retikulum, Lysosom und Nukleus umgeben.^[9]^ Beispiele für Proteine, deren Funktion durch posttranslationale Prenylierung moduliert wird, sind z. B. Mitglieder der Ras‐Superfamilie, die eine zentrale Rolle in der zellulären Signalweiterleitung spielen. Zu den häufig farnesylierten Proteinen dieser Familie gehören K‐Ras, N‐Ras, H‐Ras, Rheb, Zellkernlamina und Hdj2, während Proteine wie Rac, Cdc42, RhoA und Rab‐Proteine häufig geranylgeranyliert sind.^[14]^

Bis vor kurzem waren drei Protein‐Prenyltransferasen bekannt, die für die Prenylierung von Cysteinseitenketten in eukaryotischen Zellen verantwortlich sind. Farnesylgruppen werden von der Farnesyltransferase (FTase) transferiert, wobei Farnesylpyrophosphat (Fpp) als Substrat genutzt wird. Geranylgeranylgruppen hingegen werden durch die Geranylgeranyltransferase (GGTase‐I) übertragen, die Geranylgeranylpyrophosphat (GGpp) als Substrat nutzt.^[9]^ Beide dieser enzymkatalysierten Reaktionen finden an einem C‐terminalen CAAX‐Box‐Motiv statt. Hierbei ist C die Cysteinseitenkette, die modifiziert wird, A ist eine aliphatische Aminosäure und der X‐Rest bestimmt die Art der Modifikation. Wenn X ein Serin (Ser), Methionin (Met) oder Glutamin (Gln) ist, wird das Cys farnesyliert, wohingegen es im Falle von Leucin (Leu) geranylgeranyliert wird.[^15^, ^16^] Es gibt auch Hinweise darauf, dass die FTase kleine hydrophobe Reste an der zweiten A‐Position präferiert.^[17]^ Nach der Prenylierung werden die restlichen Aminosäuren (AAX) der Box abgespalten. Dies geschieht entweder durch eine Protease des endoplasmatischen Retikulums oder durch das Ras‐konvertierende Enzym 1. Das entstehende Carboxylat des prenylierten Cys wird anschließend durch das Enzym Isoprenylcystein‐Carboxymethyltransferase (ICMT) methyliert, sodass ein C‐terminaler Methylester entsteht.^[18]^ Im Gegensatz zu den FTase‐ und GGTase‐I‐Prenyltransferasen gibt es eine dritte Klasse an Enzymen, die Rab (Ras‐related in brain)‐Geranylgeranyltransferase (GGTase‐II oder Rab GGTase), die GGpp als Substrat nutzt, um eine oder zwei Geranylgeranylgruppen zu transferieren. Die C‐terminalen Prenylierungsmotive, die in der Familie der Rab‐Proteine gefunden wurden, sind meistens CC und CXC, aber auch CCX, CCXX und CXX.[^9^, ^19^] Eine vierte Art einer Prenyltransferase mit dem Namen GGTase3 wurde erst vor kurzem entdeckt und ist für die Geranylgeranylierung der Ubiquitin‐Ligase FBXL2 verantwortlich, wodurch diese sich an die Membran anlagert und die Polyubiquitylierung von membranverankerten Proteinen ermöglicht.^[20]^ In ähnlicher Weise modifiziert es SNARE‐Proteine wie Ykt6, was eine Voraussetzung für den korrekten Aufbau des Golgi‐SNARE‐Komplexes ist.^[21]^

### 1.2 Acylierung

Myristoylierung und Palmitoylierung repräsentieren die beiden häufigsten Formen von Protein‐Acylierung, und es wurde für beide gezeigt, dass sie einen kritischen Einfluss auf die Proteinstruktur, ‐funktion und/oder ‐lokalisierung besitzen.[^9^, ^22^, ^23^] Palmitoylierung ist definiert durch die Anbringung einer C_16_‐Palmitinsäure an ein Protein, wobei im Menschen zwei unterschiedliche Verknüpfungen möglich sind (Abbildung 1). Bei der S‐Palmitoylierung wird das Lipid reversibel an die Seitenkette eines Cysteinrestes angebracht, wobei eine enzymatisch und hydrolyselabile Thioesterbindung entsteht. Bei der N‐Palmitoylierung wird das Lipid auf den N‐Terminus eines Proteins, oder seltener auf eine Lysinseitenkette, transferiert und bildet dort eine hydrolysestabile Amidbindung aus. Interessanterweise ist bis jetzt kein Enzym bekannt, das eine N‐Palmitoylierung entfernen kann, sodass diese Modifikation als irreversibel angesehen wird.^[22]^ Im Gegensatz zu vielen anderen PTMs, wie zum Beispiel bei den Prenylierungen, gibt es keine spezifische Erkennungssequenz, die eine Vorhersage von N‐Palmitoylierung erlaubt. Zusätzlich könnten einige der publizierten N‐Palmitoylierungen das Ergebnis eines *S*‐zu‐*N*‐Acyl‐Transfers sein.^[24]^ Die S‐Palmitoylierung tritt jedoch häufig in Verbindung mit nahe gelegenen N‐myristoylierten Glycin (Gly)‐Resten oder prenylierten C‐terminalen Cys‐Seitenketten auf.^[22]^

Die Palmitoylierung hat ebenfalls einen wichtigen Einfluss auf den Aufenthaltsort von Proteinen, da sie die Hydrophobie erhöht und somit zu einer Anlagerung von ansonsten löslichen Proteinen an verschiedene Zell‐ und Organellmembranen führt. In Neuronen ist Palmitoylierung wichtig für die Anlagerung von Proteinen an Axon‐Enden, wodurch die Synapsenaktivität reguliert wird.^[25]^ Bei vielen Proteinen ist S‐Palmitoylierung nicht permanent, sondern wechselt zwischen Palmitoylierung und Depalmitoylierung, um die Proteinfunktion in einer dynamischen Weise zu regulieren.^[26]^ Solche dynamischen Modifikationszyklen werden enzymatisch reguliert, wobei die Addition des Lipids durch Palmitoyltransferasen und die Entfernung durch Acylprotein‐Thioesterasen, wie Acylprotein‐Thioesterase‐1 (APT1)^[27]^ oder Palmitoylthioesterase‐1 (PPT1),^[28]^ erfolgt. Zu den am genauesten untersuchten Beispielen von S‐palmitoylierten Proteinen gehören Mitglieder der Ras‐Proteinfamilie, die zu den kleinen GTPasen gehören. Hier wurde gezeigt, dass die Lipidierung die Proteinanlagerung an Membranen beeinflusst.^[29]^ Es gibt auch Anhaltspunkte dafür, dass Palmitoylierung Proteine vor proteasomalem Abbau schützt, indem die Ubiquitylierung verhindert wird.[^30^, ^31^]

N‐Myristoylierung von Proteinen wird definiert als das Anbringen einer C_14_‐Myristoylgruppe an einen N‐terminalen Gly‐Rest eines Proteins über eine Amidbindung. In manchen Fällen können Proteine auch Myristoylgruppen an der ϵ‐Aminogruppe von Lysinen (Lys) tragen, wie beispielsweise in TNFα und in dem Interleukin‐1α‐Protein.[^32^, ^33^, ^34^] Im Gegensatz zur Prenylierung und Palmitoylierung kann Myristoylierung sowohl co‐ als auch posttranslational auftreten. Nach der Abspaltung des N‐terminalen Met‐Restes durch eine Methionin‐Aminopeptidase wird die Myristoylgruppe eingebracht.^[35]^ In Eukaryoten sind hauptsächlich die beiden *N*‐Myristoyltransferasen (NMT1 oder NMT2) dafür verantwortlich, den Transfer einer Myristoylgruppe von Myristoyl‐CoA auf ein Substratprotein, das einen N‐terminalen Gly‐Rest enthält, zu katalysieren.^[36]^ Wie andere Arten der Lipidierung ist auch die N‐Myristoylierung für die Regulierung von Signalweiterleitungen, Membranassoziation und Proteinlokalisierung verantwortlich. Weit verbreitet sind auch duale Myristoyl‐Modifikationen.[^37^, ^38^] In vielen Fällen ist eine Myristoylgruppe nicht ausreichend, um eine Membrananlagerung zu induzieren, sodass zusätzliche Lipidierungen notwendig sind, um die Hydrophobie zu erhöhen. Aus diesem Grund werden Myristoylierung und Palmitoylierung häufig zusammen in Proteinen gefunden.^[39]^

Es ist wichtig anzumerken, dass auch seltene Protein‐Fettsäure‐Acylierungen mit Octanoat (am besten bekannt als *O*‐Octanoat‐Modifikation an dem Peptidhormon Ghrelin)^[40]^ oder mit ungesättigten C_16–20_‐Fettsäureketten auftreten.

### 1.3 Glycosylphosphatidylinositol‐Anker

Die Anbringung von einem Glykosylphosphatidylinositol (GPI)‐Anker (auch Glypiation genannt) ist eine posttranslationale Modifikation, die in einem breiten Spektrum von Organismen zu finden ist, darunter Säugetiere, Insekten, Pflanzen, Pilze und Protozoen.^[41]^ Die Modifikation findet am C‐Terminus von Proteinen statt und dient als Anker für die Anlagerung an die extrazelluläre Seite von Plasmamembranen.^[41]^ Die GPI‐Struktur beinhaltet einen Phosphoethanolaminlinker, ein hochkonserviertes Glykan‐Grundgerüst (d‐Man(α1‐2)‐d‐Man(α1‐6)‐d‐Man(α1‐4)‐d‐GlcN(α1‐6)*myo*‐inositol) und einen Lipidschwanz, der abhängig von dem Organismus, aus dem er stammt, variiert. Die Länge dieser Lipide kann zwischen 14 und 28 Kohlenstoffatomen variieren, und sie können gesättigt oder ungesättigt sein.^[42]^ Während viele biologische Funktionen von GPI‐Ankern noch unklar sind, wurde schon herausgefunden, dass sie eine Hauptrolle in Zell‐Zell‐Adhäsion, Signaltransduktion, Membranadressierung und Lipid‐Rafts‐Abtrennungen spielen.[^43^, ^44^, ^45^] Bertozzi und Mitarbeiter haben zahlreiche elegante chemisch‐biologische Studien durchgeführt, um die Struktur‐Funktions‐Beziehungen von GPI‐Ankern zu untersuchen. Sie haben beispielsweise gezeigt, dass das interne Glykan des GPI wichtig für die laterale Mobilität von Proteinen ist, um die Aktivität zu regulieren.[^46^, ^47^] Durch den Einsatz einer leistungsfähigen Strategie zur Veränderung der Zelloberfläche wurden aufgereinigte, GPI‐modifizierte Proteine an Zellmembranen von exogenen Quellen verankert, sowohl in vitro als auch in vivo.[^48^, ^49^, ^50^] Es wurde auch gezeigt, dass GPI‐Anker wichtige Komponenten von immundominanten Epitopen für eukaryotische Parasiten (z. B. *Plasmodium falciparum*) sind, was dazu geführt hat, dass synthetische Varianten zur Nutzung als Impfstoffkandidaten produziert wurden.[^51^, ^52^] Für diese Studien ist es entscheidend, dass die GPI‐verankerten Peptide und Proteine in ausreichender Menge und Reinheit erhalten werden. Wenn sie durch rekombinante Expression in Zellen produziert werden, sind die Proben normalerweise heterogen, mit einer großen Strukturvariation im Lipidbereich des GPI. Diese sind durch chromatographische Methoden sehr schwer zu trennen.[^45^, ^53^] Daher hat sich die chemische Synthese als eine mögliche Route herausgestellt, um Peptide mit GPI‐Anker für funktionale Studien herzustellen. Um die komplizierte und laborintensive Synthese des nativen GPI‐Moleküls zu vermeiden, wurden einige vereinfachte Mimetika synthetisiert und untersucht. Diese werden jedoch nicht detailliert in diesem Aufsatz dargestellt.[^45^, ^54^, ^55^]

### 1.4 Cholesterol‐Anker

Cholesterol‐Anker werden im Kontext von Hedgehog (Hh)‐Proteinen gefunden, die wichtig für das embryonale Wachstum und die Bildung einer Vielzahl von Tumorarten sind.[^56^, ^57^] Diese Cholesterol‐Modifikationen werden mittels eines einzigartigen Selbstspaltungsprozesses eingebaut, bei dem die 3β‐Hydroxygruppe von Cholesterol über eine Esterbindung an den C‐Terminus des prozessierten Proteins geknüpft wird (Abbildung 1).[^56^, ^57^] Diese Reaktion wird durch einen intramolekularen Angriff von einer Cysteinseitenkette auf die Carbonylgruppe eines benachbarten Glycinrestes initiiert, wodurch als Intermediat eine Thioesterbindung entsteht. Dieser Thioester reagiert anschließend mit der 3β‐Hydroxygruppe von Cholesterol, sodass eine Esterbindung entsteht und die C‐terminale Autoprozessierungsdomäne freigesetzt wird.^[9]^ Es wurde gezeigt, dass C‐terminale Cholesterol‐Anker für die Freisetzung von doppelt lipidierten Hh‐Proteinen von der Zelloberfläche verantwortlich sind. Dies wird durch die beiden Transporter‐ähnlichen Proteine (Scube und Disp), die Teile des Cholesterol‐Moleküls erkennen, vereinfacht.[^58^, ^59^, ^60^] Es ist wichtig anzumerken, dass Cholesterol zwar für die Hh‐Signalaktivität nicht zwingend notwendig ist, aber dass die Abwesenheit dieser Modifikation die Signalstärke verringert.^[59]^ Des Weiteren ist Cholesterol wichtig für die Regulation der Aktivität des Proteins Smoothened (SMO). Dieses wird eher an einem Asparaginsäure (Asp)‐Rest als am C‐Terminus modifiziert.^[61]^

### 1.5 Phosphatidylethanolamin‐Anker

Die Addition von Phosphatidylethanolamin (PE)‐Modifikationen, um PE‐Anker zu generieren, ist eine seltene und unterstudierte PTM. Bis heute wurden PE‐Anker in den Autophagie‐bezogenen Proteinen Atg8 (Hefe) und LC3 (Säugetiere) gefunden.^[62]^ Diese Anker sind durch eine Amidbindung zwischen einem C‐terminalen Glycinrest und der Aminogruppe des PE miteinander verknüpft.^[63]^ In den Atg8‐ und LC3‐Proteinen ist die Bindung an PE essenziell für ihre korrekte Lokalisierung und Funktion.^[64]^

## 2 Methoden für die Herstellung von lipidierten Peptiden und Proteinen

### 2.1 Synthesemethoden

**Figure 2:**
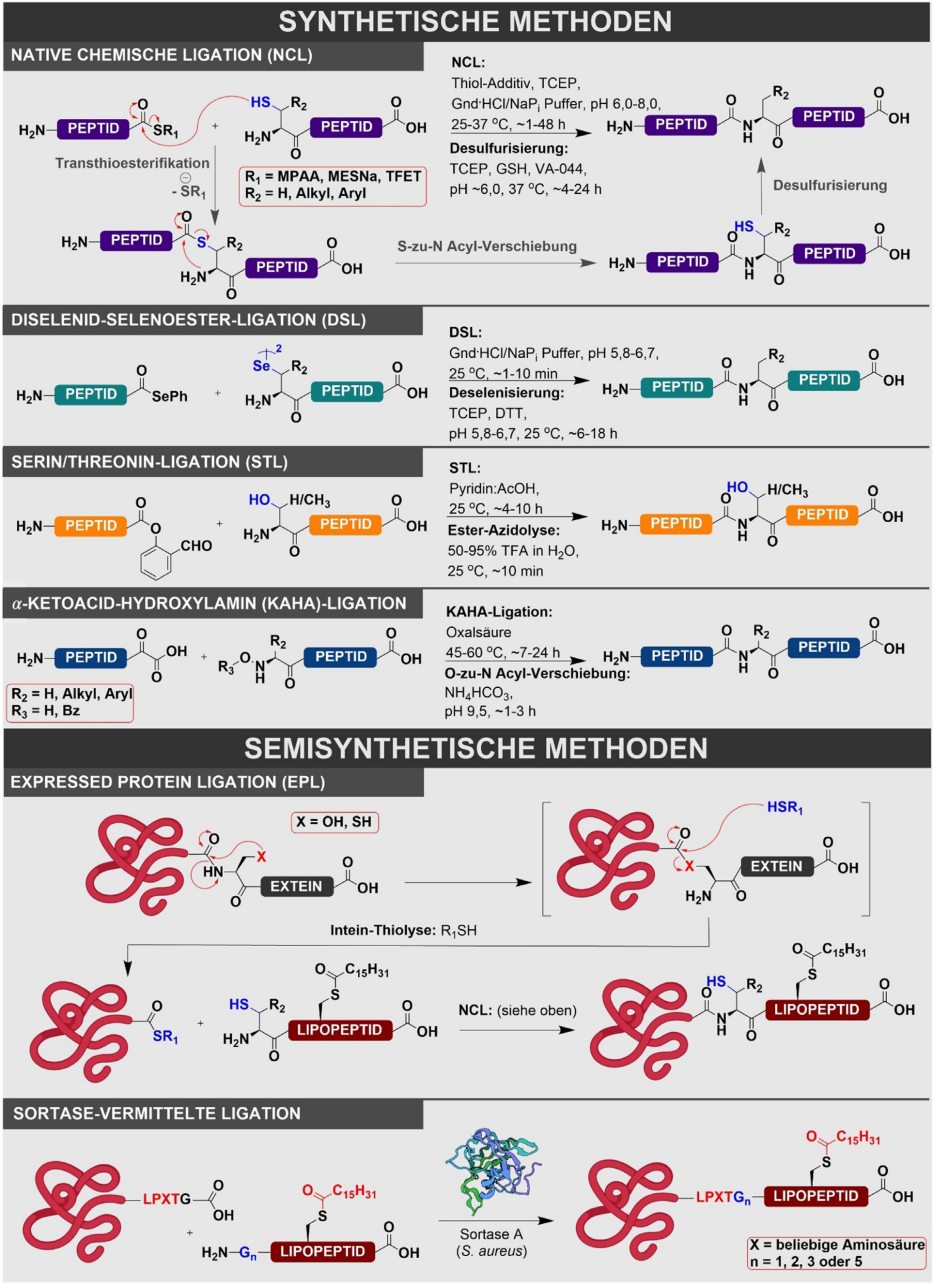
Allgemeine Übersicht über die verschiedenen Strategien für die Herstellung von lipidierten Proteinen mittels chemischer Synthese oder Semisynthese.

Im Allgemeinen können lipidierte Peptide routinemäßig mittels Festphasenpeptidsynthese (solid‐phase peptide synthesis, SPPS) hergestellt werden. Obwohl einige Strategien für die Synthese lipidierter Peptide in Lösung entwickelt wurden,[^65^, ^66^, ^67^, ^68^] sind diese Ansätze in der Regel sehr aufwendig und erfordern zahlreiche Schutzgruppenmanipulationen und Aufreinigungsschritte. Zusätzlich werden die Löslichkeitsprobleme, die mit der Verwendung seitenkettengeschützter Peptide bei der Peptidsynthese in Lösung einhergehen, bei Lipopeptiden noch verstärkt.^[64]^ Diese Probleme werden an einer festen Phase vermindert, da eine nahezu vollständige Reaktion durch einen Überschuss an Reagenzien, der durch einfache Filtration entfernt wird, erreicht werden kann. Lipidierte Peptide können durch SPPS entweder durch die Kupplung von lipidierten Aminosäuren an die wachsende Peptidkette oder durch die selektive Lipidierung von spezifischen ungeschützten Aminosäuren generiert werden.^[69]^ Die Produktion von lipidmodifizierten Proteinen ist erheblich anspruchsvoller. Dies liegt an den Größenlimitierungen der Peptidsynthese (bei SPPS‐Methoden typischerweise 40–50 Reste). Aus diesen Gründen werden lipidierte Peptide und Proteine mit einer Länge von mehr als 50 Aminosäuren üblicherweise über Ligationschemie hergestellt. Hierbei können ein Peptid und ein lipidiertes Proteinfragment chemoselektiv miteinander verknüpft werden, sodass größere Zielproteine erhalten werden können (Abbildung 2). Größere lipidierte Proteine, deren Herstellung durch chemische Totalsynthese sehr schwierig und aufwendig wäre, können durch semisynthetische Methoden produziert werden. Als Methodik wird Proteinsemisynthese im Allgemeinen kategorisiert als die Nutzung von chemischen oder chemoenzymatischen Methoden, um ein synthetisches Peptid an ein größeres und (typischwerweise) unmodifiziertes exprimiertes Protein zu fusionieren (Abbildung 2). Eine der am häufigsten genutzten Methoden ist die expressed protein ligation (EPL), die die Vielfalt der NCL ausnutzt, um die chemische Ligation von einem rekombinanten Protein mit einem synthetischen Peptid zu vereinfachen. Dies wurde zuerst durch eine Ligation von einem rekombinanten Proteinthioester (generiert durch Thiolyse von einem Protein‐Intein‐Fusionskonstrukt) und einem synthetischen Peptid mit einem N‐terminalen Cysteinrest gezeigt. Als Alternative wurde eine Vielzahl von Methoden für chemoenzymatische Semisynthese entwickelt. Die relevanteste Methode für diesen Artikel ist die Sortase‐vermittelte Ligation, bei der ein Protein, das C‐terminal mit einer Sortase‐Erkennungssequenz markiert ist, regioselektiv durch das Sortase‐Enzym (siehe unten) an ein C‐terminales Peptid oder Protein fusioniert wird (Abbildung 2).

Rekombinante Expressionsmethoden zur Proteingewinnung nutzen routinemäßig *E. coli*.[^70^, ^71^] Zusätzlich zu der einfachen genetischen Manipulation und Handhabung wächst *E. coli* schnell und stellt typischerweise große Mengen an Zielprotein her. Allerdings ist es schwierig, manche komplexen Säugetierproteine, die einen hohen Grad an struktureller Komplexität aufweisen, durch ein einfaches bakterielles Expressionssystem zu erhalten. Während Prokaryoten dafür bekannt sind, Proteine posttranslational zu modifizieren, resultiert die Nutzung von *E.‐coli*‐Expressionssystemen meistens in der Produktion von unmodifizierten Proteinen, und es ist kompliziert, bakterielle Standardexpressionssysteme für die Einbringung von Säugetier‐PTMs zu nutzen.^[71]^ Es sollte angemerkt werden, dass eukaryotische Expressionssysteme wie Hefe^[72]^ oder Insektenzellen^[73]^ die notwendige Enzymmaschinerie besitzen, um viele häufige PTMs zu generieren. Allerdings sind solche Methoden von Natur aus mit Problemen behaftet, da heterogene Mischungen von Proteinen gebildet werden. Dies schränkt ihre Anwendung für die Untersuchung der Auswirkungen bestimmter PTMs und für den Zugang zu genau definierten, ortsspezifisch modifizierten Proteintherapeutika – einer Klasse von Biomolekülen, von der vorhergesagt wird, dass sie das Fundament der sich weiter rasant entwickelnden Biotechnologie‐ und “Biologics”‐Industrie bildet – stark ein.[^64^, ^74^] Aus diesem Grund stellt die semisynthetische Herstellung von lipidierten Proteinen durch chemoselektive Ligation von einem unmodifizierten rekombinanten Protein und einem homogen lipidierten synthetischen Peptid eine enorm wichtige methodische Grundlage dar.

Die Herstellung von Volllängenproteinen ausgehend von synthetisch und/oder rekombinant hergestellten Segmenten ist mittels verschiedener Ligationsverfahren möglich (Abbildung 2).[^3^, ^75^, ^76^] Die am häufigsten genutzte Methode ist die native chemische Ligation (NCL).[^77^, ^78^] Andere Methoden sind zum Beispiel die Diselenid‐Selenoester‐Ligation (DSL),[^79^, ^80^, ^81^] Ser/Thr‐Ligation (STL),^[82]^ α‐Ketoacid‐Hydroxylamin‐Ligation (KAHA‐Ligation),^[83]^ Maleimidocaproyl‐Ligation (MIC‐Ligation)^[68]^ und Sortase‐vermittelte enzymatische Ligation.^[84]^ In den meisten Fällen werden ungeschützte Peptid‐ oder Proteinfragmente genutzt. Dies ermöglicht es, die Reaktionen in gepufferten wässrigen Lösungen mit neutralem pH (oder nahe des neutralen pH) und bei Raumtemperatur bzw. gering erhöhten Temperaturen durchzuführen. Die NCL‐Methode beinhaltet eine chemoselektive Reaktion zwischen einem Peptid mit einem N‐terminalen Cystein‐Rest und einem anderen Peptid, das als C‐terminaler Thioester derivatisiert wurde. Mechanistisch gesehen beginnt die NCL‐Reaktion mit einem nukleophilen Angriff der Cysteinseitenkette (am N‐Terminus des einen Segments) auf einen Thioester (am C‐Terminus des anderen Segments) in einem reversiblen Transthioveresterungsschritt. Diesem Schritt folgt eine schnelle *S*‐zu‐*N*‐Acyl‐Verschiebung, um eine native Amidbindung zu formen.[^77^, ^78^] Die Synthese von Peptidthioestern kann durch verschiedene Protokolle in Lösung und an der festen Phase erreicht werden.^[85]^ Größere Proteinthioester können durch die Nutzung von konstruierten Inteinen hergestellt werden, die einen natürlichen Proteinspleißprozess verwenden.^[86]^ In diesem Prozess wird ein internes Peptidfragment innerhalb eines Proteins (Intein) autokatalytisch aus dem Protein herausgeschnitten, wodurch die beiden flankierenden Segmente (Exteine) ligiert werden, indem sich eine Amidbindung ausbildet. Im ersten Schritt wird durch einen reversiblen *N*‐zu‐*S*/*O*‐Acyl‐Transfer ein (Thio)ester an einem N‐terminalen Cys‐ oder Ser‐Rest des Inteins gebildet (Abbildung 2). Dieses Intermediat wird nach einem nukleophilen Angriff durch einen Cys‐, Ser‐ oder Thr‐Rest am C‐Terminus des Exteins einer Trans(thio)veresterung ausgesetzt. Der entstehende (Thio)ester durchläuft eine intramolekulare Zyklisierung an dem konservierten Asparagin (Asn)‐Rest am C‐Terminus des Inteins. Diese Succinimid‐Bildung schneidet das Intein heraus, und nach einer finalen *S*/*O*‐zu‐*N*‐Acyl‐Verschiebung wird eine Amidbindung zwischen den beiden Exteinen gebildet.[^87^, ^88^] Im Kontext von EPL wird ein Fusionskonstrukt des Zielproteins, das mit einer Intein‐Domäne verknüpft ist, die nur die initiale Thioesterbildung durchlaufen kann, genutzt. Es können Affinitäts‐Tags wie zum Beispiel eine Chitin‐bindende Domäne (CBD) am C‐Terminus des Konstruktes angebracht werden, um die Proteinaufreinigung zu vereinfachen. Nach der Aufreinigung mittels Affinitätschromatographie kann das Fusionsprotein (durch einen Überschuss an Thiol, wie beispielsweise 2‐Mercaptoethansulfonat (MESNa)) abgespalten und von der Säule eluiert werden, wodurch der entsprechende MESNa‐Thioester des gewünschten Proteinsegments entsteht. Dieser kann dann über eine NCL‐Reaktion an ein synthetisches Lipopeptid mit einem N‐terminalen Cystein ligiert werden.[^64^, ^87^]

Die DSL‐Methodik orientiert sich an der NCL‐Reaktion, aber sie nutzt die überragende Reaktivität von C‐terminalen Selenoestern mit der erhöhten Nukleophilie der 21. Aminosäure Selenocystein (Sec) am N‐Terminus des anderen Peptidfragments. Diese erhöhte Reaktivität macht die Ligationen einfacher als NCL, sodass sie auch bei niedrigen Konzentrationen durchgeführt werden können. Aus diesem Grund ist DSL eine nützliche Ligationsmethode, wenn die Reaktionspartner eine geringe Löslichkeit aufweisen (z. B. lipidierte Fragmente), und sie wurde bereits erfolgreich für die Synthese von lipidierten Proteinen eingesetzt.^[89]^ STL beinhaltet die chemoselektive Reaktion zwischen einem ungeschützen Peptid mit einem C‐terminalen Salicylaldehyd (SAL)‐Ester und einem anderen ungeschützten Peptid mit einem N‐terminalen Ser‐ oder Threonin (Thr)‐Rest.[^90^, ^91^] Die Häufigkeit von Ser‐ und Thr‐Resten in nativen Proteinen macht dies zur einer attraktiven Methode für die Proteinsynthese. Ein finaler Typ der Peptidligationsreaktionen, die in diesem Aufsatz abgedeckt werden, ist die α‐Ketoacid‐Hydroxylamin (KAHA)‐Ligation.[^83^, ^92^] Diese Reaktion erfolgt über die decarboxylierende Kondensation eines Peptides mit einem α‐Ketoacid am C‐Terminus mit einem Peptid, das eine N‐terminale Hydroxylamin‐Funktionalität trägt. Dies ermöglicht die Fusionierung der Fragmente über eine Amidbindung, üblicherweise unter sauren Bedingungen in einem Gemisch aus Wasser und organischen Lösungsmitteln. Obwohl diese Schlüsseltechniken bereits vorhanden sind, ist davon auszugehen, dass zukünftige Innovationen, die auf diesen Peptidligationskonzepten beruhen, die Zahl der lipidierten Proteine, die durch Synthese und Semisynthese zugänglich sind, weiter erhöhen werden.

Zwei weitere Methoden, die genutzt wurden, um lipidmodifizierte Proteinanaloga zu generieren, sind die Maleimidocaproyl (MIC)‐ und Sortase‐Ligationen. Für die MIC‐Ligation wird das synthetische Lipopeptid mit einer N‐terminalen Maleimidgruppe ausgestattet,^[68]^ die über eine Michael‐Addition mit dem Seitenkettenthiol eines Cys‐Rests in einem exprimierten Proteinfragment reagiert. Ein Vorteil dieser Methode ist, dass Cys‐Reste am C‐Terminus mit einem gewissen Grad an Selektivität modifiziert werden können, da die restlichen Cys innerhalb der Sequenz sterisch unzugänglich sind, wenn sie im gefalteten Protein verborgen sind.^[93]^ Allerdings ist diese Methode nicht mit multiplen C‐terminalen Lys‐Resten kompatibel, da die ϵ‐Amin‐Funktionalität auch mit der elektrophilen Maleimid‐Einheit reagieren kann.^[94]^ Im Gegensatz dazu profitiert die Sortase‐Ligation davon, dass sie die Fähigkeit des Sortase‐Enzyms, spezifisch ein LPXTG‐Pentapeptidmotiv [X=vorzugsweise Ser oder Glutamat (Glu)] zu erkennen, nutzt und daher chemoselektiv zwei Peptid‐ oder Proteinfragmente fusionieren kann (Abbildung 2).^[95]^ Nach der Bindung an das Erkennungsmotiv initiiert die Sortase die Thiolyse der T‐G‐Amidbindung, sodass ein Thioester‐verknüpftes Acyl‐Enzym‐Intermediat entsteht. Dieser Thioester wird von dem α‐Amin eines N‐terminalen Gly‐Restes eines anderen Peptid‐ oder Proteinfragments abgefangen. Hierbei entsteht das Transpeptidierungsprodukt und das aktive Enzym wird regeneriert.[^84^, ^95^] Ein großer Nachteil dieser Strategien ist, dass sie “Narben” in der Proteinsequenz hinterlassen, z. B. ein unnatürliches Maleimid für MIC‐Ligationen und ein LPXTG‐Motiv für Sortase‐Ligationen. Andere Ligations‐ oder Biokonjugationstechniken, die erfolgreich genutzt worden sind, um unnatürliche Analoga oder Mimetika von lipidierten Proteinen zu erhalten, beinhalten die Diels‐Alder‐Reaktion^[96]^ und die Cu^I^‐katalysierte Azid‐Alkin‐Cycloaddition (CuAAC).^[97]^

### 2.2 Methoden zur Verbesserung der Handhabung und Analyse von lipidierten Peptiden und Proteinen

Die Einbringung von Lipiden in Peptide und Proteine kann einen sehr starken Einfluss auf ihre physikochemischen Eigenschaften haben, was zu schlechter Löslichkeit und daraus folgend zu Aggregation und der Bildung von Mizellen in wässrigen Puffern führen kann.^[98]^ Es wurden schon viele Versuche unternommen, um die Löslichkeit von lipidierten Peptiden und Proteinen zu erhöhen, indem Pufferadditive wie Detergenzien und/oder Chaotrope zugegeben oder kurzzeitig Löslichkeits‐Tags eingebracht wurden. Die verbesserte Löslichkeit, die durch diese Strategien hervorgerufen wird, hilft sowohl bei Ligations‐ und Lipidmodifizierungsreaktionen als auch bei der Handhabung während der Aufreinigung.

In den meisten Fällen werden lipidierte Peptide und Proteine mittels Umkehrphasen‐Hochleistungsflüssigkeitschromatographie (reverse phase high performance liquid chromatography, RP‐HPLC) analysiert, da die gesteigerte Hydrophobie eine einfache Abtrennung von unmodifizierten und/oder kleineren Vorstufen ermöglicht. Hier wird die Nutzung von kurzkettigen stationären Phasen mit ausreichender Porengröße empfohlen, z. B. eine C_4_‐Säule mit einer Porengröße von 30 nm. Die Nutzung von Säulen mit längeren Alkylketten, wie beispielsweise C_18_, verkompliziert die Aufreinigung durch eine erhöhte Affinität der Lipidmodifikation zu diesen stationären Phasen.^[99]^ Die Wahl des organischen Eluenten und Additivs für RP‐HPLC sowie die Temperatur sind wichtige weitere Parameter. Es ist wichtig anzumerken, dass normalerweise für jedes Protein (oder zumindest für verschiedene Proteinklassen) individuelle optimierte Aufreinigungsbedingungen identifiziert werden müssen. Außerdem muss sichergestellt werden, dass die Nutzung eines niedrigen pH und/oder hoher Temperaturen während der Aufreinigung nicht zu einer Zersetzung des Proteins oder zur Abspaltung der Lipidketten führt.^[100]^

In vielen Fällen hilft die Zugabe von mit Wasser mischbaren organischen Lösungsmitteln, wie beispielsweise Acetonitril oder fluorierte Alkohole, um lipidierte Peptide und Proteine vor und während der Aufreinigung löslicher zu machen. Fluorierte Alkohole wie 2,2,2‐Trifluorethanol (TFE) oder 1,1,1,3,3,3‐Hexafluor‐2‐propanol (HFIP) sind starke Wasserstoffbrückenbindungendonoren und können α‐helikale Sekundärstrukturen stabilisieren, was dabei hilft, Peptide und Proteine mit helikalen Strukturelementen in Lösung zu behalten. Kombinationen aus organischen Lösungsmitteln (z. B. Acetonitril mit 2‐Propanol und TFE) mit geringen Konzentrationen an TFA als Additiv wurden erfolgreich für die Aufreinigung von lipidierten Proteinen mittels RP‐HPLC eingesetzt.^[101]^

Auch wenn es noch bessere Lösungsmittel gibt, um hydrophobe Peptide und Proteine zu lösen, wie zum Beispiel Dimethylformamid (DMF) und Dimethylsulfoxid (DMSO), werden diese nicht standardmäßig eingesetzt, da sie eine starke Absorption bei 214 nm aufweisen (ähnlich zu der Absorption der Peptidbindung) und somit bei der HPLC‐Aufreinigung mit der Absorption von Peptiden und Proteinen interferieren.^[102]^ Die Zugabe von DMSO kann auch zur Oxidation von Methionin‐ und Cysteinseitenketten führen.^[103]^ Falls keine Bedingungen für RP‐HPLC identifiziert werden können, stellt die hydrophobe Interaktionschromatographie (hydrophobic interaction chromatography, HIC) eine alternative Methode für die Aufreinigung dar. Für diese Methode wird eine hydrophobe stationäre Phase (z. B. Agarose mit Butyl‐ oder Phenyl‐Liganden) genutzt und die Proben werden in einem Puffer mit hoher Salzkonzentration auf die Säule geladen. Ein abnehmender Salz‐Gradient wird genutzt, um die Proteine nach steigender Hydrophobie von der Säule zu eluieren.^[104]^ Es wird angemerkt, dass HIC nicht für die Aufreinigung von Peptiden und Proteinen geeignet ist, die nur über hydrophobe Wechselwirkungen Aggregate bilden.

In solchen Fällen werden häufig wässrige Puffer mit hohen Konzentrationen von Chaotropen, wie beispielsweise Guanidinium‐Hydrochlorid (Gdn⋅HCl, bis 6 M) oder Harnstoff (bis 8 M), in Kombination mit Detergenzien genutzt, um der Aggregation von hydrophoben Peptiden und Proteinen vorzubeugen, indem Wasserstoffbrückenbindungsdonoren und ‐akzeptoren gestört und hydrophobe Interaktionsstellen mit Detergenzmolekülen blockiert werden.[^105^, ^106^] Über der kritischen Mizellkonzentration (critical micelle concentration, CMC) bilden Detergenzien eine ideale Umgebung für Lipide. Allerdings können diese auch zur Denaturierung von Proteinen führen, und sie sind oft schwierig durch Standardaufreinigungsmethoden zu entfernen. Die am häufigsten genutzten Detergenzien sind das negativ geladene Natriumdodecylsulfat (sodium dodecyl sulfate, SDS) sowie nicht‐ionische Detergenzien wie Triton X‐100 und Tween 20. Es ist wichtig anzumerken, dass die Polyethylenglykol‐Bestandteile in Triton X‐100 und Tween 20 gut ionisieren und dadurch bei der Analyse mittels Massenspektrometrie die Signale von anderen Molekülen unterdrücken können. Aus diesem Grund werden häufig andere Detergenzien wie beispielsweise Octylglykosid (OG) und *n*‐Dodecyl‐β‐D‐maltosid (DDM) bevorzugt, da sie einfach mittels Dialyse oder durch die Nutzung von Biobeads entfernt werden können.^[102]^

In Fällen, in denen die oben beschriebenen Strategien (oder Kombinationen davon) nicht zu erfolgreichen Ligationen und/oder Aufreinigungen führen, ist eine andere Methode die kovalente Anbringung von Löslichkeits‐Tags, die nach dem Ligations‐ oder Faltungsschritt wieder entfernt werden können. Solche kurzzeitig angebrachten Moleküle verkomplizieren die Synthese und können durch zusätzliche Arbeitsschritte die Ausbeute verringern, aber sie können signifikant die Rückgewinnung von hydrophoben Peptiden und Proteinen durch RP‐HPLC erhöhen. Eine der bereits sehr früh publizierten Strategien beinhaltete die Einführung von Polylysin‐Tags (fünf oder mehr Lysinreste) am N‐ oder C‐Terminus von Transmembranproteinen und wurde erfolgreich eingesetzt, um die Löslichkeit von Peptiden, die von dem humanen Erythrocyt‐Protein Glycophorin A, Bakteriophage‐M13‐Hüllenprotein und dem Hepatitis‐C‐Virus‐Membranprotein NS4A abgeleitet sind, in wässrigen Puffern zu erhöhen.^[107]^ Allerdings waren die ersten Versionen von Löslichkeits‐Tags permanent angebracht, und aufgrund ihrer hohen Ladungsdichte konnten sie die biologische Funktion von Peptiden und Proteinen beeinflussen. Daraus resultierend wurden temporäre Löslichkeits‐Tags, wie Polyethylenglykolpolyamid, Polyethylenglykol und Polyarginin, entwickelt. Diese können an den Termini, an den Seitenketten oder am Polypeptidrückgrat angebracht werden. Hierbei existiert eine Vielzahl an verschiedenen Linkern, die eine Abspaltung der Tags unter sauren^[108]^ oder basischen^[109]^ Bedingungen oder durch die Nutzung von spezifischen Proteasen erlauben.[^110^, ^111^] Es wurden auch Löslichkeits‐Tags entwickelt, die über photolabile Linker verknüpft wurden, um potenziell schädliche Reaktionsbedingungen bei der Abspaltung zu vermeiden.^[112]^ Basierend auf der oben dargestellten Zusammenfassung sind zwar einige Strategien verfügbar, um die Handhabung und Reinigung von lipidierten Peptiden und Proteinen zu verbessern, aber es gibt keine allgemeingültige Strategie, und in einigen Fällen werden möglicherweise mehrere dieser Ansätze ausprobiert werden müssen. Im Allgemeinen ist festzuhalten, dass, wenn kein Puffer gefunden werden kann, der die Löslichkeit erhöht und andere Alternativen wie die Nutzung von Chaperonen (siehe Abschnitt 6 für lipidierte Rab‐Proteine) nicht verfügbar sind, Löslichkeits‐Tags am Rückgrat des Zielpeptids/‐proteins eine gute Option sind, um homogen lipidierte Proteine handhabbar zu machen.

## 3 Synthese und Semisynthese von palmitoylierten Peptiden und Proteinen

S‐palmitoylierte Lipopetide können mittels Fmoc‐Strategie‐SPPS (mit angepassten Fmoc‐Entschützungsbedingungen, um Thioesterhydrolyse und *S*‐zu‐*N*‐Acyl‐Transfer zu vermeiden) erhalten werden, indem lipidierte Aminosäurebausteine genutzt werden. Ein Beispiel für diese Strategie ist die Synthese der Harz‐gebundenen endothelialen Stickstoffmonoxid‐Synthase (eNOS)_1–26_ (**1**) in der Waldmann‐Arbeitsgruppe (Schema 1 A).^[113]^ Alternativ kann die Einbringung der Lipidmodifikation in das Substratpeptid in einer späteren Phase der Synthese erfolgen, wie gezeigt bei der palmitoylierten Variante des Matrixproteins M2_31–96_ (**2**, Schema 1 B).^[114]^ N‐palmitoylierte Lipopeptide sind sehr einfach herzustellen, indem Palmitinsäure während der SPPS unter Standardkupplungsbedingungen direkt an den N‐Terminus eines Peptides kondensiert wird. Dies wurde bei der Herstellung des N‐terminalen Fragments des Sonic Hedgehog Peptids (ShhN)_1–34_ (**3**) gezeigt (Schema 1 C).^[115]^ Ein bekanntes Problem bei der Festphasensynthese von S‐palmitoylierten Peptiden und Proteinen ist die Labilität gegenüber Standard‐Fmoc‐Entschützungsbedingungen (z. B. 20 vol. % Piperidin in DMF), wobei *S*‐zu‐*N*‐Acyl‐Verschiebungen stattfinden können, wenn sich ungeschützte Cysteinreste am N‐Terminus befinden.^[116]^ Um dieser ungewollten *S*‐zu‐*N*‐Acyl‐Verschiebung vorzubeugen, kann während der Peptidsynthese eine Fmoc‐Entschützungslösung mit 1 vol. % DBU in DMF genutzt werden, direkt gefolgt von dem nächsten Aminosäurekupplungsschritt.^[116]^ Ein häufiges Problem, das bei der Synthese von Peptiden mit Fettsäuremodifikationen auftritt, ist die geringe Löslichkeit, die zur Bildung von Aggregaten führt. Dieses Problem wird verstärkt durch die Tatsache, dass die amphiphatische Natur von Lipopeptiden zu einem breiteren Elutionsprofil an der stationären Phase, die für die chromatographische Aufreinigung (z. B. HPLC) genutzt wird, führt. Dies macht es schwieriger, Nebenprodukte während der Aufreinigung zu entfernen. Aus diesem Grund ist es normalerweise notwendig, mehrere Peptid‐ und Lipopeptidfragmente mittels Ligationschemie zu einem Protein zusammenzufügen oder transiente Solubisierungs‐Tags zu nutzen, wie sie auch bei der Synthese von Membranpeptiden und ‐proteinen eingesetzt werden.^[117]^ In diesem Kontext werden häufig alternative Ligationsstrategien, wie beispielsweise STL, DSL und direkte Aminolyse, genutzt, da Palmitoylthioester in Gegenwart von Thioladditiven, die genutzt werden, um die Geschwindigkeit von NCL‐Reaktionen zu erhöhen, instabil sind.[^118^, ^119^, ^120^]

**Scheme 1:**
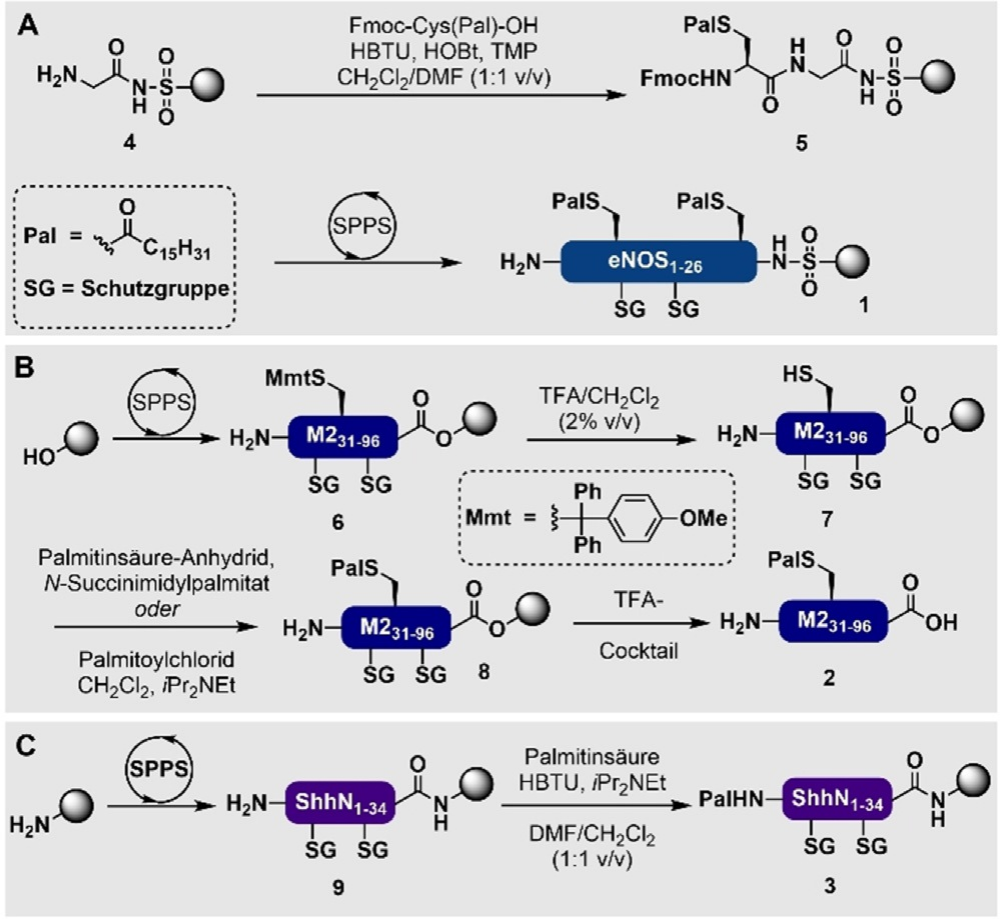
Festphasensynthese von S‐palmitoylierten Peptiden mittels A) Kupplung von lipidierten Aminosäuren^[113]^ oder B) direkter Palmitoylierung von ungeschützten Cystein‐Resten am Harz.^[114]^ C) Festphasensynthese von N‐palmitoylierten Peptiden durch direkte Kupplung von Palmitinsäure an den N‐Terminus.^[115]^ SG=Standard‐Seitenkettenschutzgruppen, die in der Fmoc‐SPPS genutzt werden.

Bis heute wurden zahlreiche palmitoylierte Proteine durch chemische Totalsynthese erhalten. Palà‐Pujadas et al. haben eine beeindruckende NCL‐Strategie basierend auf fünf Segmenten mit kinetischer Kontrolle genutzt, um die palmitoylierte N‐terminale Domäne des humanen Sonic Hedgehog Proteins mit 175 AS herzustellen.^[115]^ Hirabayashi und Mitarbeiter haben die Totalsynthese und strukturelle Charakterisierung des Caveolin‐1 (**10**, 178 AS), welches in der C‐terminalen Region an Cys_133_, Cys_143_ und Cys_156_ dreifach S‐palmitoyliert ist, durchgeführt (Schema 2 A).^[120]^ Retrosynthetisch wurde das Protein in fünf Peptidsegmente unterteilt, die in vier aufeinanderfolgenden Ligationsreaktionen zusammengefügt wurden. Die Synthese wurde mit einer direkten Aminolyse zwischen den Isopeptidfragmenten **11** und **12** begonnen, sodass das Intermediat **13** gebildet wurde. Anschließend folgten iterative Ligationsreaktionen, sodass die komplett geschützte Caveolin‐1‐Primärsequenz (**14**) erhalten wurde. Die chemoselektive Acm‐Entschützung, die Palmitoylierung der drei ungeschützten Cys‐Reste [durch das elektrophile *N*‐Succinimidylpalmitat (Pal‐OSu)‐Reagenz] und die globale Entschützung führten zu dem lipidierten Zielprotein **10** (Schema 2 A). Es ist wichtig anzumerken, dass die Gruppe feststellte, dass die von Caveolin‐1 abgeleiteten Peptide sehr unlöslich in wässrigen Puffern waren. Daher wurden *O*‐Acyl‐Isopeptidbindungen eingebaut, um die Löslichkeit der Segmente zu verbessern. Nach der Herstellung des gesamten Proteins wurden sie wieder in native Peptidbindungen umgewandelt. Zusätzlich, um wässrige Lösungsmittel zu umgehen, hat die Gruppe direkte Aminolysereaktionen in DMSO genutzt (statt traditioneller Ligationsverfahren), um die Fragmente zu verknüpfen. Diese Bedingungen sind vorteilhaft für die Stabilität von Palmitoylthioestern, da diese nicht über einen längeren Zeitraum in wässrigen Puffern stabil sind. Trotzdem ist die direkte Aminolyse nicht die typische Methode der Wahl, da sie potenziell zu 1) Regioselektivitätsproblemen, wenn andere nukleophile Reste in dem Peptidfrragment vorhanden sind (z. B. Lys‐ und Cys‐Reste), und 2) Epimerisierung an Cα der Ligationsstelle während der Aktivierung des C‐terminalen Restes von einem der Fragmente führen kann. Regioselektivitätsprobleme können vermieden werden, indem passende Schutzgruppen an Lys‐ und Cys‐Resten genutzt werden, und Epimerisierung wird unterdrückt, indem Ligationsstellen gewählt werden, die nur Gly‐, Pro‐ oder Ser‐Reste an der N‐terminalen Seite besitzen.^[120]^

**Scheme 2:**
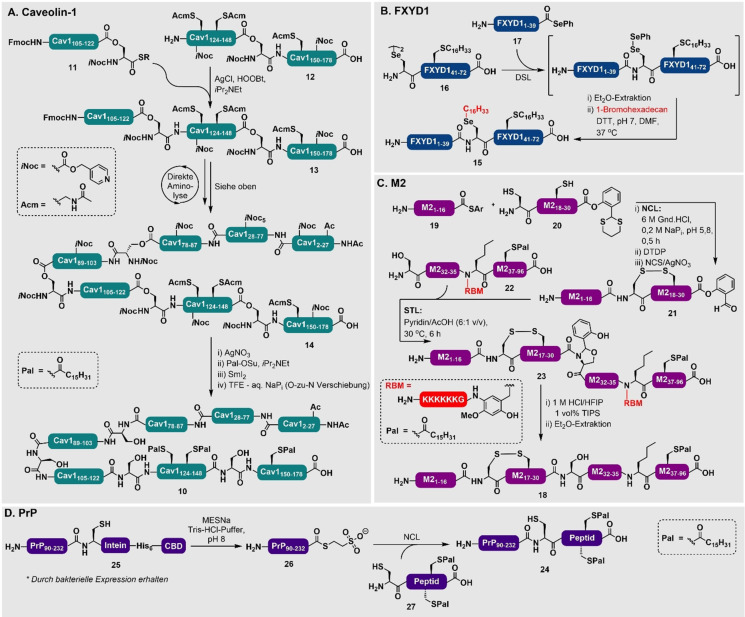
A) Direkte Aminolyse‐basierte Synthese von palmitoyliertem Caveolin‐1 (**10**) von Hirabayashi und Mitarbeitern.^[120]^ B) Synthese eines lipidierten FXYD1 (**15**) durch Tandem‐DSL‐Alkylierung von Chisholm et al.^[89]^ C) Synthese des palmitoylierten M2‐Ionenkanals (**18**) von Huang et al. unter Nutzung eines RBM‐assistierten STL‐Ansatzes.^[118]^ D) Semisynthese eines palmitoylierten PrP (**24**) von Becker und Mitarbeitern.[^55^, ^123^]

In einem anderen Beispiel haben Chisholm et al. das Membranprotein Phospholemman (FXYD1)_1–72_ (**15**) erhalten, indem sie “reduktive DSL”‐Chemie mit geringen Konzentrationen genutzt haben. Hierdurch wurde die Löslichkeit des Lipopeptidfragments **16**, das einen palmitoylierten Cys_42_‐Rest enthält, erreicht (Schema 2 B).^[89]^ Nach der DSL‐Reaktion zwischen **16** und einem FXYD1_1–39_ N‐terminalen Selenoester **17** wurde der Sec‐Rest an der Ligationsstelle (Sec_40_) mit 1‐Bromhexadecan alkyliert, um ein dipalmitoyliertes Analogon des Proteins zu erhalten.^[89]^ Hanna et al. benutzten eine Vier‐Komponenten‐DSL/NCL‐Strategie, um di‐ und tripalmitoylierte Varianten des *Mycobacterium‐tuberculosis*‐assoziierten Antigen‐Proteins ESAT6 zu synthetisieren.^[121]^ Hierbei wurde die Palmitoylierung am N‐terminalen Ligationsfragment durch eine direkte Kupplung der lipidierten Aminosäure erhalten. Dieses Fragment wurde später über eine Strategie der Seitenkettenverankerung in einen C‐terminalen Thioester umgewandelt.^[122]^ Es wurde eine Kombination aus DSL/NCL‐Chemie genutzt, um einen Großteil des ESAT6_17–95_‐Proteins herzustellen. Dieses wurde anschließend in einer letzten NCL‐Reaktion an die palmitoylierte N‐terminale Sequenz fusioniert.

In einem letzten Beispiel für die synthetische Vielfalt haben Huang et al. einen entfernbaren Rückgratmodifizierungs‐Tag (removeable backbone modification, RBM tag) in Kombination mit STL‐Chemie genutzt, um die Löslichkeit eines Lipopeptidfragments für die Synthese des S‐palmitoylierten Sarcolipin (SLN) und S‐palmitoylierten Influenza‐A‐Virus‐Matrix‐2 (M2)_1–96_ (**18**)‐Ionenkanalproteins zu erhöhen. Der in dieser Studie genutzte RBM‐Tag (eine 2‐Hydroxy‐3‐methoxy‐4‐amidobenzyl‐Gruppe, die einen 4‐Amidohexalysin‐Teil für die Löslichkeit enthält), hebt den Nutzen von Löslichkeits‐Tags bei der Synthese von palmitoylierten Peptidfragmenten hervor.^[119]^ Insbesondere die STL‐Methode ist für die Synthese dieser Zielstrukturen attraktiv, da die Reaktion, im Gegensatz zur NCL, keine Thioladditive benötigt, die den Palmitoylthioester angreifen würden. Um dies auszuweiten, haben Huang et al. eine Methode entwickelt, um sequenzielle NCL‐STL‐Reaktionen in N‐zu‐C‐Richtung zu ermöglichen. Diese Methode wurde genutzt, um den gleichen S‐palmitoylierten M2‐Ionenkanal (**18,** Schema 2 C), zusammen mit einem S‐palmitoylierten Interferon‐induzierten Transmembranprotein 3 (S‐palm IFITM3) zu synthetisieren.^[118]^ Die Synthese von **18** erfolgte durch eine NCL‐Reaktion zwischen dem Thioester **19** und einem Cysteinyl‐Fragment **20** (mit einem maskierten SAL‐Ester). Eine C‐terminale Aktivierung durch eine Behandlung mit *N*‐Chlorsuccinimid (NCS)/AgNO_3_ führte zu dem SAL‐Aktivester‐Intermediat **21**, das mit dem hydrophoben Serinylfragment **22**, das den RBM‐Tag enthält (um die Löslichkeit zu erhöhen, siehe oben), unter STL‐Bedingungen zu der M2_1–96_‐Vorstufe **23** reagierte. Das Ziel‐M2‐Protein **18** wurde durch eine finale Azidolyse des SAL‐Oxazolidins erhalten.

Semisynthetische Ansätze sind sehr gut geeignet, um größere lipidmodifizierte Proteine zu erhalten. Ein Hauptvorteil ist die Vermeidung von multiplen Ligationsschritten, die normalerweise notwendig sind, wenn Proteine mit mehr als 100 Aminosäuren allein durch chemische Synthese hergestellt werden. Während semisynthetische Methoden für die Herstellung einer Vielzahl von unterschiedlich modifizierten Proteinen genutzt wurden, gibt es aber nur eine begrenzte Anzahl an Beispielen für palmitoylierte Proteine. Erwähnenswert ist hier die Erzeugung von palmitoylierten Maus‐Prionprotein (PrP)‐Varianten (**24**) durch die Nutzung einer EPL‐Strategie (Schema 2 D).[^55^, ^123^] Diese semisynthetische Strategie beruht auf der Expression eines rekombinanten murinen PrP_(90–232)_‐Fragments **25**, fusioniert mit einem *Mxe*‐GyrA‐Mini‐Intein und zwei Affinitäts‐Tags (His_6_‐Tag und Chitin‐bindende Domäne).

Ein Überschuss von Mercaptoethansulfonat‐Natriumsalz (MESNa) wurde genutzt, um die Ser‐Cys‐Amidbindung zwischen dem Intein und dem Rest des rPrP‐Proteins zu spalten, sodass der MESNa‐Thioester **26** gebildet wurde. Dieser wurde dann in einer Thiophenol‐vermittelten Ligationsreaktion mit dem palmitoylierten Peptid‐Membrananker‐Fragment **27** umgesetzt, sodass das lipidierte Zielprotein **24** entstand. Durch die Nutzung dieser Strategie wurden fünf verschiedene palmitoylierte Varianten mit durchschnittlich 30 % Ausbeute hergestellt. Diese wurden genutzt, um den Einfluss von lipidiertem PrP auf die Membranstruktur und die Proteinverteilung in der Membran zu untersuchen.^[124]^ Dieser Ansatz wurde später auf die Konstruktion von PrP‐Varianten mit N‐terminalen Verkürzungen oder Deletionen ausgeweitet.^[123]^

## 4 Synthese von myristoylierten Peptiden und Proteinen

Die N‐terminale Myristoylierung wird normalerweise durch die direkte Kupplung einer Fettsäure oder eines aktivierten Fettsäureesters an das N‐terminale Amin eines geschützten Peptides während der SPPS erreicht. Da sie relativ leicht zu synthetisieren sind, gibt es eine große Anzahl von Beispielen für kurze, myristoylierte Peptide, die synthetisch produziert wurden.[^125^, ^126^, ^127^] Beispielsweise haben Waldmann und Mitarbeiter eNOS_1–26_, das N‐myristoyliert und an zwei Positionen S‐palmitoyliert ist, synthetisiert, indem sie eine Kombination aus orthogonalen enzymlabilen, säurelabilen und edelmetalllabilen Schutzgruppen in einem Fragmentkondensationsansatz genutzt haben.^[128]^ Allerdings war diese Methode sehr arbeitsintensiv und die Ausbeuten waren gering (<1 %), was dazu führte, dass die Gruppe einen linearen Festphasenansatz entwickelte, bei dem der Ellman‐Sulfonamid‐Harz‐Linker genutzt wurde (Schema 3 A).^[113]^ Die Gruppe erweiterte ihre Festphasensynthese von Harz‐gebundenem palmitoyliertem eNOS_1–16_ (**1**, Schema 1 A) um eine finale Fmoc‐Entschützung und Myristoylierung mittels Myristoylchlorid (Myr‐Cl) am Harz, um die Harz‐gebundene Vorstufe **28** in voller Länge zu erhalten. Nach der Alkylierung des Harz‐Linkers (mit Iodacetonitril) sowie anschließender Abspaltung und Aufreinigung ergab diese Strategie das myristoylierte und dipalmitoylierte eNOS_1–26_‐Peptid **29** in einer stark verbesserten Ausbeute von 24 %. Es ist wichtig anzumerken, dass durch diese Strategie 25 mg des eNOS‐Peptids **29** synthetisiert werden konnten, und zwar in einem Zeitraum von Tagen bis Wochen anstatt Monaten.

**Scheme 3:**
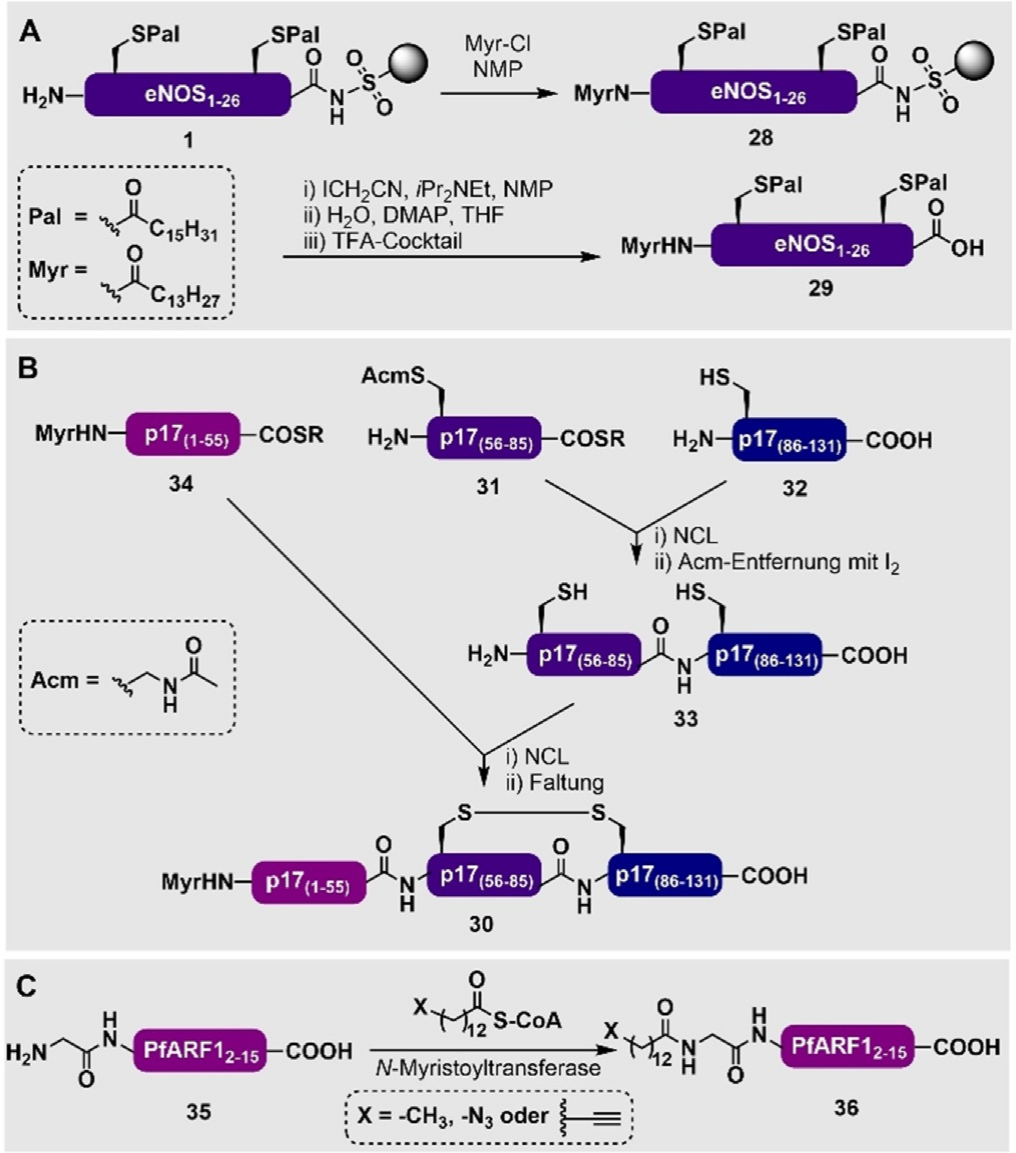
A) Synthese des myristoylierten und palmitoylierten eNOS_1–26_ (**29**) durch eine von Waldmann und Mitarbeitern entwickelte Methode an der festen Phase.^[113]^ B) Synthese von Myristoyl‐p17_1–131_ (**30**) durch einen NCL‐Ansatz mit drei Fragmenten von Lu und Mitarbeitern.^[129]^ C) Metabolische Einbringung von *N*‐Myristoyl‐Analog in PfARF1_1–15_ (**36**) von Tate und Mitarbeitern.^[130]^

Der Zugang zu größeren myristoylierten Proteinen wird typischerweise durch Semisynthese oder Mehrkomponenten‐Ligationsstrategien ermöglicht. Ein Beispielprotein, das durch chemische Synthese erschlossen wurde, ist das 131‐Reste‐N‐myristoylierte HIV‐1‐Matrix‐Protein p17_1–131_ (**30**), welches von Lu und Mitarbeitern durch eine konvergente NCL‐Strategie mit drei Segmenten erhalten wurde (Schema 3 B). Zur Herstellung der einzelnen Peptidfragmente hat die Gruppe eine In‐situ‐Neutralisierungsmethode genutzt, die von Kent und Mitarbeitern für SPPS mittels Boc‐Strategie entwickelt wurde.^[129]^ Zuerst wurde das Acm‐geschützte Thioesterfragment **31** an das C‐terminale Cysteinylfragment **32** ligiert, um den nicht‐lipidierten C‐Terminus des Proteins (**33**) zu erhalten. Eine finale NCL‐Reaktion mit dem myristoylierten N‐terminalen Fragment **34**, derivatisiert als Thioester, gab dann das finale Myristoyl‐p17‐Protein (**30**). Es ist wichtig anzumerken, dass die Erschließung dieses synthetisch myristoylierten HIV‐1‐Matrix‐Proteins **30** es den Autoren ermöglichte, die “Myristoyl‐Schalter”‐Hypothese zu untersuchen, die sich auf die Fähigkeit des Proteins bezieht, mit der Zellmembran in reversibler Weise zu interagieren.

Es wurden auch chemoenzymatische und metabolische Ansätze genutzt, um *N*‐Myristoyl‐Analoga, die z. B. mit terminalen Azid‐ oder Alkinfunktionalitäten ausgestattet sind, zu erhalten. Als Beispiel dient hier die enzymatische Myristoylierung von PfARF1_1–15_ (**35**), die Tate und Mitarbeiter durchgeführt haben, um sowohl myristoylierte als auch Azido‐myristoylierte Derivate des PfARF1_1–15_ (**36**) zu produzieren (Schema 3 C).[^130^, ^131^, ^132^] Diese Reaktionen werden von *N*‐Myristoyltransferasen katalysiert, die das N‐terminale GXXXS‐Motiv erkennen und dadurch die Fluoreszenzmarkierung und Untersuchung von myristoylierten Proteinen in Zellen ermöglichen. Diese Methoden wurden bereits zusammengefasst und werden hier nicht weiter diskutiert.^[133]^

## 5 Synthese von prenylierten Peptiden

Wie bei anderen lipidierten Peptiden basiert auch die Herstellung von prenylierten Peptiden auf Fmoc‐SPPS‐basierten Protokollen. Das Lipid kann entweder direkt am Harz eingebracht werden, indem prenylierte Aminosäurebausteine genutzt werden,^[134]^ oder alternativ können unmodifizierte Peptide nach der Harzabspaltung in Lösung alkyliert werden.[^135^, ^136^, ^137^, ^138^] Die Reaktivität der Prenylgruppe und ihre Tendenz zu isomerisieren können während der Synthese zu einigen Herausforderungen führen. Beispielsweise ist die Alken‐Funktionalität unter sauren oder reduzierenden Bedingungen nicht stabil, sodass säurelabile oder hydrogenolytisch labile Harz‐Linker oder Schutzgruppen nicht für die Synthese von prenylierten Peptiden eingesetzt werden können.^[116]^ Des Weiteren müssen die Prenylgruppen mit den Kupplungsbedingungen des lipidierten Cystein‐Bausteins kompatibel sein.^[116]^ Da Cys zu Racemisierung neigt, wurden die Kupplungsbedingungen intensiv untersucht und optimiert. Eine 1:1‐Mischung aus HBTU/HOBt oder HCTU mit Trimethylpyridin (TMP) als Base in CH_2_Cl_2_/DMF (1:1 v/v) minimiert die Racemisierung bei Kupplung an die feste Phase.[^64^, ^139^, ^140^]

Eine weit verbreitete Strategie für die Synthese von prenylierten Peptiden basiert auf der Nutzung von sehr säurelabilen 2‐Chlortritylchlorid (2‐CTC)‐Linkern. Dieser Ansatz ermöglicht die Abspaltung des Lipopeptides vom Harz unter schwach sauren Bedingungen [z. B. 1 vol. % TFA oder fluorierte Alkohole wie Trifluorethanol (TFE) oder Hexafluor‐2‐propanol (HFIP)], die mit den Prenylmodifikationen kompatibel sind. Der einzige Nachteil dieser Strategie ist, dass die Abspaltung vom 2‐CTC‐Harz eine C‐terminale Carbonsäure erzeugt, wohingegen die meisten prenylierten Proteine natürlicherweise einen C‐terminalen Methylester aufweisen. Günstigerweise können die Peptide stattdessen auch über die Seitenkette einer Aminosäure am Harz angebracht werden, die eine funktionalisierbare Seitenkette besitzt und dann die Kupplung eines passenden Aminosäuremethylesters an den C‐Terminus ermöglicht. Mithilfe dieser Methode haben Waldmann und Mitarbeiter den Methylester des farnesylierten K‐Ras‐Peptides 4B (**37**) hergestellt. Hierbei sind sie mit einem Seitenketten‐verknüpften Fmoc‐Lys‐OAll (**38**) (Schema 4 A) gestartet.[^141^, ^142^] Ausgehend davon führten Allylester‐Entschützung und anschließende Kupplung von H_2_N‐Cys(Far)‐OMe zu dem Harz‐gebundenen Lipopeptid **39**. Dieses farnesylierte Dipeptid wurde mittels Fmoc‐SPPS verlängert, um das farnesylierte K‐Ras 4B (**40**) am Harz zu erhalten. Anschließend führte eine azidolytische Abspaltung zu dem Zielpeptid **37** mit einer Gesamtausbeute von 11 %.

**Scheme 4:**
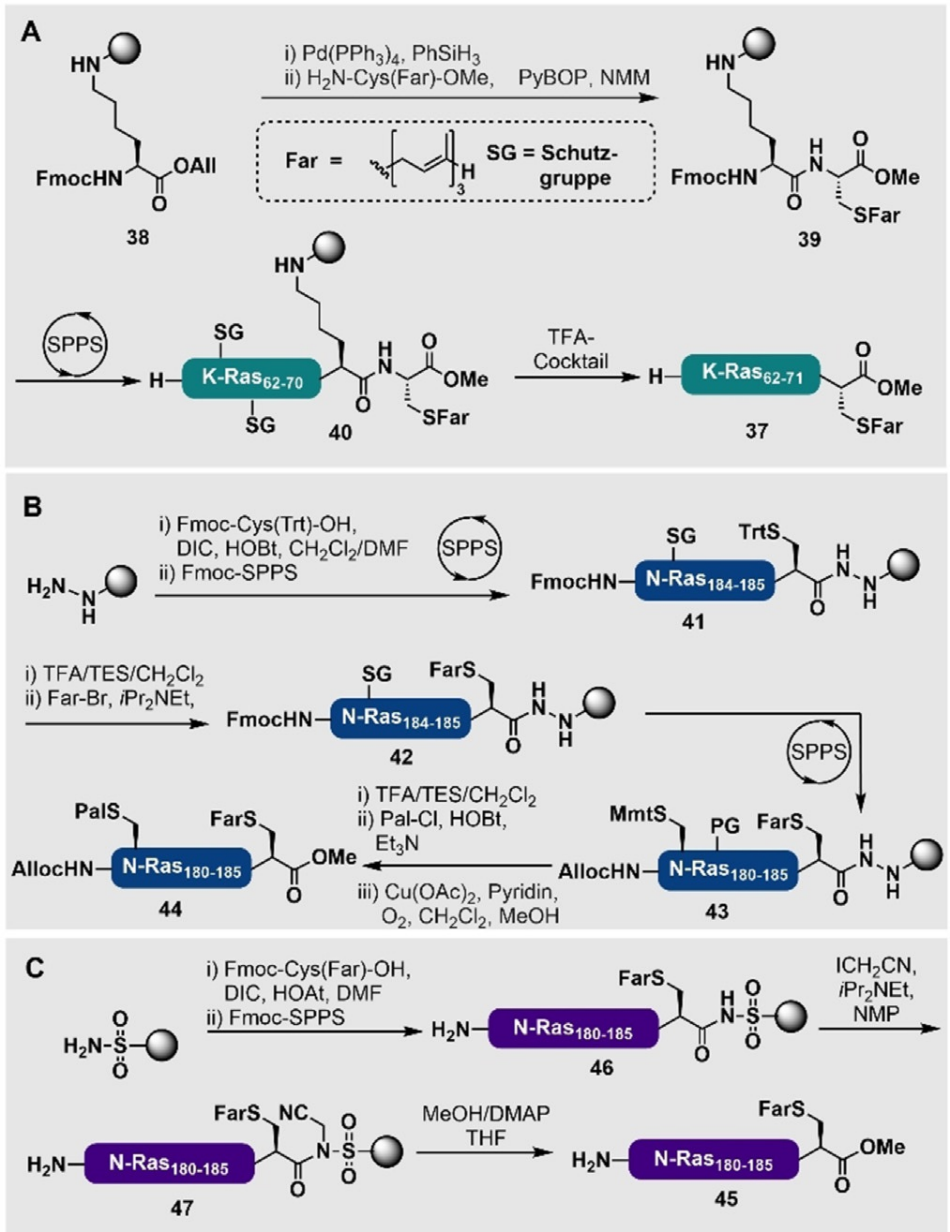
A) Synthese des farnesylierten K‐Ras‐4B‐Peptides (**37**) mit Methylester mittels einer Seitenketten‐Verankerungsstrategie durch direkte Kupplung des C‐terminalen lipidierten Restes;^[142]^ B) Nutzung eines Acylhydrazid‐Linkers, um verschieden palmitoylierte und farnesylierte N‐Ras_180–186_‐Methylester (**44**) an einem oxidativ labilen Harz‐Linker zu synthetisieren;^[136]^ C) Nutzung des Ellman‐Sulfonamid‐Linkers, um nach Alkylierung und Abspaltung farnesylierte N‐Ras_180–186_‐Methylester (**45**) zu erhalten.^[113]^

Ein alternativer Ansatz, um C‐terminale Methylester zu generieren, ist die Nutzung von oxidationslabilen Hydrazid‐Linkern, die eine Abspaltung des Peptides orthogonal zur Entfernung der Schutzgruppen erlauben, die in der Standard‐Fmoc‐SPPS verwendet werden. Auf diese Weise haben Waldmann und Mitarbeiter als erstes gezeigt, dass es möglich ist, zwei unterschiedliche Lipidierungen in ein Peptid einzubringen. Hierfür wurde eine säurestabile feste Phase genutzt, sodass das Cys(Trt) im Intermediat **41** durch schwach saure Bedingungen selektiv entschützt und anschließend mit Farnesylbromid alkyliert werden konnte, um **42** zu erhalten (Schema 4 B).^[136]^ Die Verlängerung des Peptides mittels Fmoc‐SPPS sowie die Kupplung eines Cys mit orthogonaler Monomethoxytrityl (Mmt)‐Seitenkettenschutzgruppe führten zu dem Harz‐gebundenen Intermediat **43**, das selektiv entschützt und mit Palmitoylchlorid acyliert wurde. Eine finale oxidative Abspaltung vom Harz in Anwesenheit von Methanol ergab das palmitoylierte und farnesylierte N‐Ras_180–186_ (**44**) mit C‐terminalem Methylester. Der Ellman‐Sulfonamid‐Linker findet auch Anwendung in der Herstellung von Lipopeptidthioestern, die Prenyl‐ oder Fettsäuregruppen enthalten. Der Vorteil dieses Linkers ist in diesem Fall, dass er sowohl Säuren als auch Basen gegenüber sehr stabil ist und selektiv alkyliert werden kann (z. B. mit Iodacetonitril), um ihn für die Abspaltung zu aktivieren. Die Alkylierung des Sulfonamids macht die Carbonyleinheit elektrophil, sodass diese mit Nukleophilen zur C‐terminalen Peptidmodifikation umgesetzt werden kann. Im Falle des farnesylierten N‐Ras_180–186_ (**45**) haben Waldmann und Mitarbeiter gezeigt, dass nach der Synthese des Harz‐gebundenen Farnesyl‐Peptides **46** und anschließender Linker‐Alkylierung mit Iodacetonitril, um **47** zu erhalten, Methanol genutzt werden konnte, um das Zielprotein N‐Ras_180–186_ mit C‐terminalem Methylester (**45**) zu generieren (Schema 4 C).[^113^, ^116^] Es sollte angemerkt werden, dass unerwünschte Alkylierungsreaktionen an anderen Positionen des Peptides auftreten können, wenn der Sulfonamid‐Linker alkyliert wird. Dies kann zu verringerten Ausbeuten führen. Es wurde gezeigt, dass ähnliche Strategien auch für die Synthese von geranylgeranylierten Peptiden, durch Alkylierung von passenden Peptidsubstraten mit Geranylgeranylhalogeniden, genutzt werden können.^[143]^

In einem alternativen Ansatz wurde gezeigt, dass Aziridin‐2‐carbonsäuren mittels SPPS in Peptidsequenzen eingebracht werden können. Anschließend können positions‐ und stereoselektive Öffnungen mit passenden Thiolnukleophilen, wie beispielsweise Farnesylthiol, am Harz durchgeführt werden, um prenylierte Peptide zu generieren. Diese elegante Methode wurde von Gin und Mitarbeitern angewendet, indem *N*‐Fmoc‐Aziridin‐2‐carbonsäure (Fmoc‐Azy‐OH) mittels SPPS in ein Harz‐gebundenes Tripeptid **48** eingebaut wurde, um das Pentapeptid **49** zu erhalten (Schema 5).^[144]^ Das Abfangen der Aziridinfunktionalität mit Farnesylthiol, unter basischen Bedingungen vor der Harzabspaltung und Entschützung, lieferte das S‐farnesylierte Peptid **50**. Allerdings muss diese Methode noch an größeren peptidischen Systemen getestet und die Anwendbarkeit in Anwesenheit von allen proteinogenen Aminosäuren (z. B. Cys‐Reste, die mit der Aziridin‐Gruppe reagieren können) gezeigt werden.

**Scheme 5:**
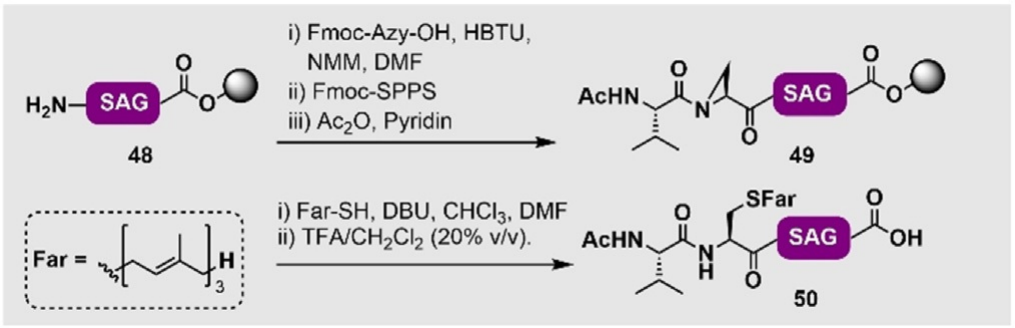
Aziridin‐vermittelte Alkylierung am Harz von Gin and Mitarbeitern.^[144]^

## 6 Synthese und Semisynthese von prenylierten Proteinen

Eine Gruppe von prenylierten Proteinen, die intensiv untersucht worden ist, gehört zu der Ras‐Superfamilie von GTPasen. Viele wichtige biologische Studien wurden durch den Zugang zu homogenen Versionen dieser modifizierten Proteine mittels semisynthetischer Methoden ermöglicht.^[116]^ Die Ras‐Superfamilie gehört zur Klasse der monomeren G‐Proteine, die in viele zelluläre Prozesse, wie die Signaltransduktion und Zellzyklusregulation, involviert sind.^[145]^ Sie können zwischen einem aktiven, GTP‐gebundenen Zustand und einem inaktiven, GDP‐gebundenen Zustand wechseln. In ihrer aktiven Form können sie durch Interaktion mit einer Vielzahl an Effektoren zelluläre Prozesse stimulieren oder inhibieren.^[64]^ Da sie wichtige molekulare Schalter sind, ist die Fehlregulierung von Ras‐Proteinen hochrelevant für die Entstehung von Krebs.^[146]^ Aus diesem Grund sind die Gene, die für diese Proteine codieren, einige der wichtigsten menschlichen Onkogene^[147]^ und konstitutiv aktive Ras‐Proteine wurden in 30 % aller soliden humanen Tumoren gefunden.[^64^, ^148^] Die am besten studierten Proteine der Familie sind die drei Ras Isoformen K‐Ras, N‐Ras und H‐Ras,^[116]^ die in den ersten 168 Resten ungefähr 90 % Sequenzidentität aufweisen. Die größten Variationen bestehen in der C‐terminalen Region (20 Reste), die auch die posttranslationalen Lipidierungen enthält.^[146]^ Die drei am häufigsten vorhandenen Typen von Lipidierung in Rab‐Proteinen sind S‐Palmitoylierung, S‐Prenylierung and N‐Myristoylierung. Diese Lipidmodifikationen führen zu der Anlagerung von Ras‐Proteinen an Membranen und sind essenziell für ihre Funktion.

Durch die Schwierigkeiten, die bei der Isolierung von lipidierten Ras‐Proteinen in voller Länge auftreten, wurden viele frühere strukturelle und biochemische Studien nur mit der löslichen Domäne, ohne den unstrukturierten, lipidierten C‐Terminus, durchgeführt.^[116]^ Um den Einfluss von Prenylierung auf die Aktivität von Ras‐Proteinen besser zu verstehen, haben einige Gruppen verschiedene Methoden entwickelt, um nativ modifizierte Ras‐Proteine zu erschließen. In den frühen 2000ern haben sich die Waldmann‐ und Kuhlmann‐Gruppen auf die Nutzung einer expressed protein MIC ligation zwischen einem exprimierten N‐Ras_1–181_‐Protein **51** (mit einem C‐terminalen Cys) und einem lipidierten Maleimidylpeptid **52** fokussiert, um zum ersten Mal palmitoyliertes und farnesyliertes N‐Ras_1–181_ (**53**) mit einem nicht‐nativen Maleimid‐Linker herzustellen (Schema 6 A).[^149^, ^150^] Ungeachtet des nicht‐nativen Linkers konnten diese Mimetika effizient in künstliche Membranen eingebracht werden und zeigten Affinität für Effektorproteine in vivo.[^68^, ^151^] Des Weiteren wurden diese semisynthetischen Proteine genutzt, um den Palmitoylierungszyklus von N‐Ras in Zellen zu untersuchen.^[26]^ Das N‐Ras wurde auch mit einem photoaktivierbaren Geranylbenzophenon‐Analogon der Farnesylmodifikation ausgestattet, das genutzt wurde, um Protein‐Protein‐ und Protein‐Lipid‐Interaktionen in Zellen zu untersuchen.^[151]^ Nach den wegweisenden Studien von Kuhlmann, Bastiaens und Waldmann zur Nutzung der MIC‐Ligation[^68^, ^152^] haben die Gruppen von Goody und Waldmann die erste Synthese eines nativ geranylgeranylierten Rab7‐Proteins durch eine Intein‐vermittelte EPL‐Strategie veröffentlicht.^[153]^ Hierfür nutzten sie ein rekombinantes Rab7‐Proteinsegment, das C‐terminal mit einem Intein fusioniert war und durch Inkubation mit MESNa gespalten wurde, um den entsprechenden Thioester zu erhalten. Dieses Rab7‐Thioestersegment wurde mit einem synthetischen, geranylgeranylierten *N*‐Cysteinylpeptid verknüpft. Diese sehr leistungsfähige EPL‐Strategie wurde seitdem genutzt, um geranylgeranylierte Ypt1‐GTPase,^[154]^ prenyliertes Rab7,^[143]^ mono‐/digeranylgeranyliertes Rab7,^[155]^ farnesyliertes Rheb und K‐Ras4B[^94^, ^156^] und farnesylierte Rheb‐Proteine zu generieren.^[157]^ In den meisten dieser Fälle war es entscheidend, ein geeignetes Detergenz zu finden, sodass die prenylierten Peptide und Proteine in wässrigen Puffern solubilisiert bleiben und die Ligationen mit ausreichenden Reaktionsgeschwindigkeiten ablaufen.[^143^, ^158^] Es sollte angemerkt werden, dass bis heute hauptsächlich Rab‐Proteine mittels EPL‐Ansätzen als C‐terminal lipidierte Proteine hergestellt wurden. Dies liegt hauptsächlich daran, dass für diese Proteine Löslichkeits‐erhöhende Bindungspartner vorhanden sind, wie beispielsweise das Rab escorting protein REP‐1, das genutzt werden kann, um entstehende lipidierte Proteine während der Ligation und der Faltung zu solubilisieren. Die Faltung ist der letzte und kritische Schritt, um aktive, posttranslational modifizierte semisynthetische Proteine zu generieren. Zwei andere solubilisierende Chaperon‐ähnliche Proteine, nämlich der GDP‐dissociation inhibitor (GDI)^[154]^ und die β‐Untereinheit der RabGGTase, wurden genutzt, um das geranylgeranylierte Rab7‐Protein^[159]^ zu renaturieren. Obwohl einige Proteine vom Ras‐Typ, darunter K‐Ras4B und D‐Ral, mithilfe der EPL‐Strategie zugänglich gemacht wurden,^[141]^ ist ein Chaperon für viele andere Mitglieder der Ras‐Proteinfamilie nicht verfügbar, was die Faltung in die native Form nach der Ligation erschwert.

**Scheme 6:**
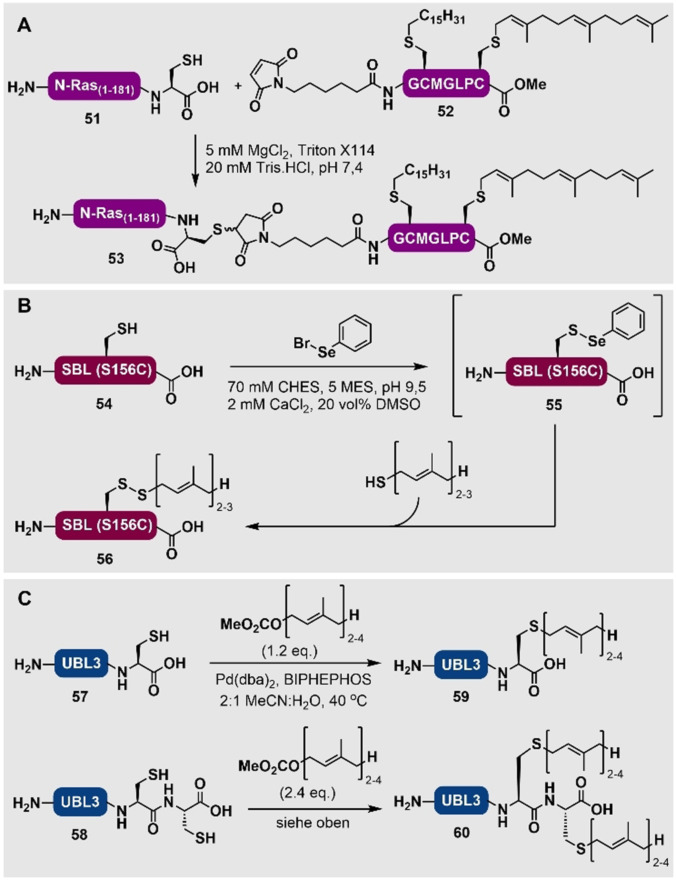
A) MIC‐basierte Ligation eines farnesylierten N‐Ras‐Analogons **53** von Waldmann und Mitarbeitern.^[149]^ B) Prenylierung einer Cysteinyl‐Subtilisin‐*Bacillus‐lentus*‐(SBL)‐Mutante **54** durch ein intermediäres Protein‐Selenylsulfid **55** von Davis und Mitarbeitern.^[162]^ C) Pd‐katalysierte Tsuji‐Trost‐Allylierung für die einfache oder doppelte Prenylierung von UBL3 [mit einem (**57**) oder zwei (**58**) C‐terminalen Cys‐Resten] von Becker und Mitarbeitern.^[163]^

Ein anderer Weg, um nativ prenylierte Proteine zu erhalten, ist die chemoselektive Modifikation von Proteinen. Trotz des Fortschrittes in diesem Bereich gibt es noch Herausforderungen zu überwinden, z. B. in Bezug auf Regio‐ und Chemoselektivität, Stabilität des entstehenden Proteins sowie die Entwicklung von Reaktionen, die in wässrigen Puffern, bei physiologischem pH und Temperatur funktionieren, um die Denaturierung des Zielproteins zu verhindern.[^160^, ^161^] Aus diesem Grund gibt es weniger Beispiele für prenylierte Proteine, die über Modifikationen des gesamten Proteins als über ligationsbasierte Strategien hergestellt wurden.^[160]^

Ein Beispiel für eine Lipidierung eines Proteins in voller Länge wurde von Davis und Mitarbeitern veröffentlicht und nutzte die einzigartige Reaktivität von Selenylsulfiden für die thiolselektive Prenylierung von Proteinen.^[162]^ Die Autoren waren in der Lage, eine S156C‐Mutante des Modellproteins Subtilisin aus *Bacillus lentus* (SBL, **54**), das ein freiliegendes Cys als Phenylselenylsulfid **55** enthält, durch eine Reaktion mit Phenylselenylbromid zu aktivieren (Schema 6 B). Diese Spezies wurde dann in wässriger Lösung (mit 20 vol. % DMSO, um die hydrophoben Prenylthiole zu lösen) mit einem prenylierten Thiol umgesetzt, was zur Bildung des asymmetrischen disulfidverknüpften Prenyl‐Proteinkonstrukts **56** führt. In ähnlicher Weise wurden sowohl Farnesyl‐ als auch Geranyl‐Modifikationen von Subtilisin erzielt (mit >50 % und >90 % Umsatz). Es sollte angemerkt werden, dass es nicht möglich war, mit dieser Methode Geranylgeranylmodifikationen einzubringen. Dies kann durch die geringe Löslichkeit des Geranylgeranylthiols in wässrigen Puffern erklärt werden, die für die Löslichkeit des Proteins notwendig sind. Ein Nachteil dieser Methode ist die unnatürliche Disulfidverknüpfung zu der Lipidmodifikation, die unter reduzierenden Bedingungen gespalten werden kann.^[162]^ Vor kurzem haben Becker, Breinbauer und Mitarbeiter eine Methode für die Prenylierung von exprimierten Proteinen mittels Pd‐katalysierter Tsuji‐Trost‐Allylierung entwickelt (Schema 6 C). Durch diese Strategie konnten die Autoren Farnesyl‐, Geranyl und Geranylgeranylgruppen sowie andere unnatürliche Modifikationen an C‐terminale Cys‐Reste des Ubiquitin‐like Proteins 3 (UBL3) einbringen. Hierbei wurden Proteinvarianten mit einem (**57**) oder zwei (**58**) Cys‐Resten genutzt, sodass das einfach (**59**) oder doppelt (**60**) lipidierte UBL3 erhalten wurde. Es ist wichtig anzumerken, dass die durch die Tsuji‐Trost‐Allylierung eingebrachten Prenylmodifikationen eine native Thioetherbindung aufweisen, sodass diese Methode eine vielversprechende Strategie für die Gewinnung von nativen Prenylmodifikationen in Peptide und Proteine in Lösung darstellt.^[163]^

## 7 Synthese von PE‐modifizierten Peptiden und Proteinen

Die synthetische Modifikation des C‐Terminus von Peptiden und Proteinen mit Phosphatidylethanolamin (PE) stellt, durch die hohe Hydrophobie der PE‐Einheit, eine große Herausforderung dar. Ein wichtiges Beispiel, welches das Löslichkeitsproblem adressiert, wurde von Liu und Mitarbeitern publiziert, die in der Lage waren, das PE‐modifizierte LC3‐II‐Protein (**61**) in großen Mengen zu erhalten.^[164]^ Der Schlüssel zu ihrem Erfolg war die Einführung eines photolabilen Löslichkeits‐Tags in ein orthogonal geschütztes Harz‐gebundenes Hexapeptid **62**. Nach schwach saurer Harzabspaltung wurde das entstehende Intermediat **63** unter DIC/HOAt‐Bedingungen an 1,2‐Distearoylphosphatidylethanolamin (DSPE) gekuppelt, sodass **64** erhalten wurde. Eine finale NCL zwischen diesem Cysteinyl‐PE‐modifizierten Hexapeptid und einem exprimierten LC3‐II‐MESNa‐Thioester (**65**; generiert durch Inteinthiolyse) ermöglichte nach einer UV‐mediierten Entfernung des photolabilen Löslichkeits‐Tags den Zugang zu dem PE‐modifizierten Zielprotein **61** (Schema 7).

**Scheme 7:**
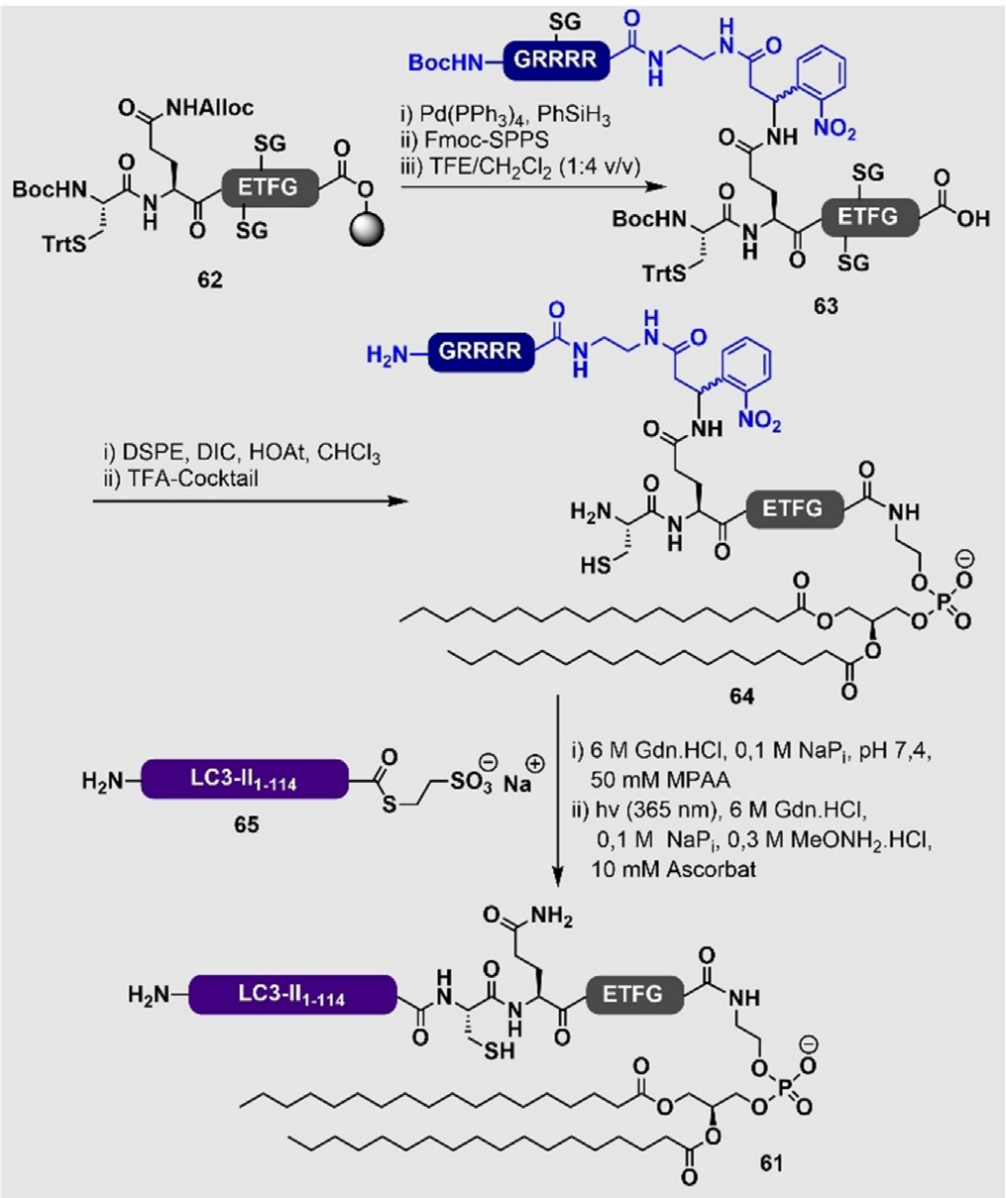
Synthese des PE‐modifizierten LC3‐II‐Proteins (**61**) von Huang et al. unter Nutzung eines photoabspaltbaren Arg‐reichen Löslichkeitspeptides an der Gln‐Seitenkette (gezeigt in Blau).^[164]^ DSPE=1,2‐Distearoylphosphatidylethanolamin.

Im gleichen Jahr wie das oben genannte Beispiel haben Wu und Mitarbeiter eine andere Semisynthese eines PE‐modifizierten LC3‐Proteins durch eine ähnliche EPL‐Strategie veröffentlicht, und das erhaltene lipidmodifizierte Protein wurde genutzt, um Autophagie zu untersuchen.^[165]^ In diesem Fall wurde das LC3‐Protein als ein N‐terminales MBP‐ und C‐terminales Intein‐Fusionskonstrukt in *E. coli* exprimiert. Die Behandlung mit MESNa ergab den MBP‐LC3‐Thioester mit ausreichender Löslichkeit, um eine Ligation an ein PE‐modifiziertes Peptid zu ermöglichen. Um das native lipidierte LC3 in voller Länge zu erhalten, wurde das MBP durch eine TEV‐Protease abgespalten. Das semisynthetisch lipidierte Protein erwies sich durch seine Interaktion mit der Protease Atg4B und seine Aktivität bei der Membranbindung und ‐fusion, die für die Rolle von LC3 in der Autophagie entscheidend sind, als funktionell.^[166]^

## 8 Synthese von Cholesterol‐modifizierten Peptiden und Proteinen

Die C‐terminale Modifikation von Proteinen mit einem Cholesterol‐Molekül ist verantwortlich für die Steuerung der Lokalisierung von Proteinen an der Zellmembran. Ein bekanntes Beispiel sind die Proteine der Hedgehog‐Familie, die ein solches C‐terminales Cholesterol aufweisen.^[167]^ Waldmann und Mitarbeiter haben Mimetika dieser Cholesterol‐modifizierten Hedgehog‐Proteine generiert, indem sie eine MIC‐Ligationsstrategie für die Verknüpfung eines exprimierten Proteinfragmentes und einem kleineren synthetischen Peptid mit einer C‐terminalen Cholesterol‐Einheit genutzt haben. Obwohl diese einen unnatürlichen Maleimidyllinker besitzen, ermöglichten die Konstrukte den Autoren die Durchführung von Schlüsselexperimenten, die zeigten, dass Cholesterol allein in der Lage ist, Proteine mit einer Affinität an Membranen zu verankern, die mit dualen Lipidierungsmotiven (z. B. S‐Farnesylierung mit zusätzlicher Geranylgeranylierung oder S‐Palmitoylierung wie in anderen lipidierten Proteinen) vergleichbar ist.^[171]^

Teruya et al. haben eine Semisynthese von GFP mit einer C‐terminalen Cholesterol‐Einheit als Modellsystem veröffentlicht. Um dieses zu erhalten, hat die Gruppe einen GFP‐Thioester mittels Intein‐Technologie erstellt. Dieser wurde an ein Peptid mit einer C‐terminalen Cholesterol‐Einheit ligiert, wobei ein Detergenz genutzt wurde, um die Löslichkeit des Lipopeptides zu erhöhen. Anschließend haben sie die Lokalisierung des Proteins an Membranen mittels konfokaler Fluoreszenzmikroskopie untersucht.^[172]^ In einer Bausatzmethode haben Blixt und Mitarbeiter ausgehend von Cholesterol (**66**) ein Azid‐enthaltendes Cholesterol‐Derivat **67** für die Reaktion mit einer Alkinyl‐Aminosäure **68** synthetisiert, um eine Triazol‐verknüpfte Aminosäure **69** zu generieren (Schema 8 A). Die Anwendbarkeit dieses cholesterylierten Bausteins **69** wurde durch die Kupplung des Bausteins an ein Harz gezeigt, was zu **70** führte und durch Standard‐Fmoc‐SPPS verlängert werden konnte, um das glykosylierte Modell‐Lipopeptid **71** zu erhalten.^[168]^ Ingallinella et al. haben eine Methode gewählt, um Cholesterol (**66**) durch eine Reaktion in Lösung zu derivatisieren. Die Reaktion von Cholesterylbromid (**72**) mit einem C34‐Peptid **73**, das ein C‐terminales Cys besitzt, führte zu cholesteryliertem C34 (**74**) (Schema 8 B). Dies erlaubte den Autoren, die antivirale Wirksamkeit von HIV‐1‐Peptidfusionsinhibitoren zu erhöhen, indem sie auf das Zellkompartiment ausgerichtet wurden, in dem die Fusion stattfindet.^[169]^

**Scheme 8:**
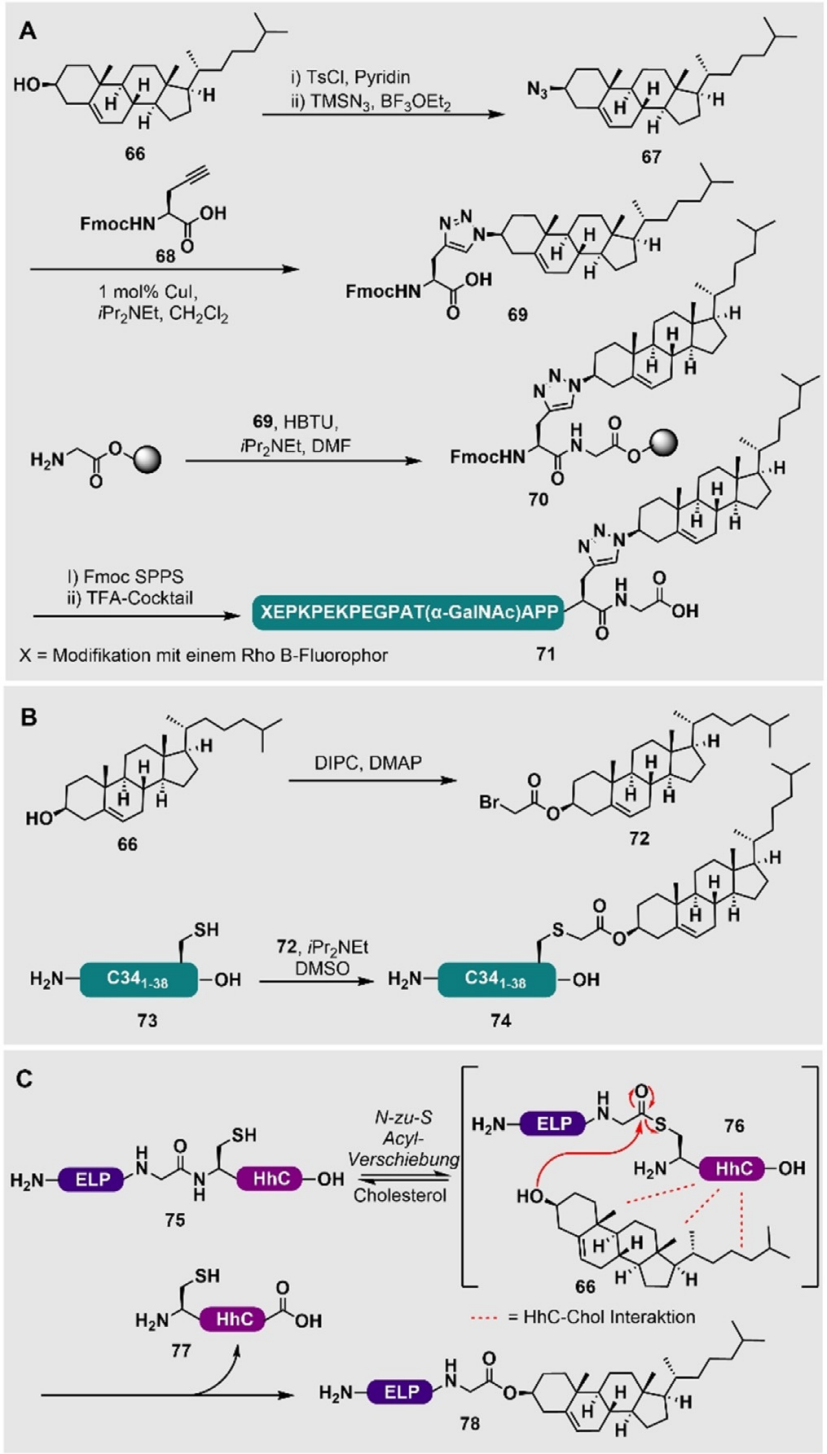
A) Synthese und Einbringung einer Fmoc‐geschützten Triazol‐verknüpften Cholesteryl‐Aminosäure (**69**) von Blixt und Mitarbeitern.^[168]^ X=weitere Modifikation mit Rho‐B‐Fluorophor. B) Derivatisierung des C34‐Peptids (**73**) mit Cholesterol von Ingallinella et al.^[169]^ C) Semisynthese eines C‐terminal Cholesterol‐modifizierten Elastin‐ähnlichen Polypeptids (ELP) (**78**) von Chilkoti und Mitarbeitern durch Fusion von ELP an ein Cholesterol‐bindendes HhC‐Protein (**75**).^[170]^

Vor kurzem haben Chilkoti und Mitarbeiter eine elegante enzymatische Methode entwickelt, um C‐terminal Cholesterol‐modifizierte Peptide und Proteine, wie beispielsweise das Elastin*‐*like Polypeptid (ELP), zu erschließen.^[173]^ Diese Strategie beinhaltete die Fusion von ELP an ein sekundäres HhC‐Protein (C‐terminale Autoprozessierungsdomäne des Hedgehog‐Proteins; **75**), das Cholesterol erkennt und bindet. Bei der Bindung von Cholesterol (**66**) wird durch eine Intein‐ähnliche *N*‐zu‐*S*‐Acyl‐Verschiebung, an der die HINT‐Domäne beteiligt ist, die das Hedgehog‐Protein mit den Inteinen teilt, ein reaktives Thioester‐Intermediat **76** gebildet. Dieser Thioester reagiert anschließend mit der 3β‐Hydroxygruppe an einem assoziierten und proximalen Cholesterol‐Molekül (**66**), was schließlich zur Extrusion der HhC‐Domäne (**77**) und Bildung des Cholesterol‐modifizierten EPL (**78**) führt (Schema 8 C).^[170]^ Die Autoren nutzten diesen Ansatz, um Cholesterol an das bioaktive Peptid Exendin‐4 anzuhängen, ein zugelassenes Peptid‐Medikament für Typ‐II‐Diabetes. Es ist wichtig anzumerken, dass die Autoren zeigten, dass die Cholesterol‐Modifikation zur Selbstorganisation der Peptide in Micellen führte, was wiederum den glucagon‐like peptide I receptor mit hoher Wirksamkeit aktivierte. Da Cholesterol in der Lage ist, Biomoleküle an bestimmte Stellen der Membran, einschließlich geordneter Bereiche (Rafts), zu leiten, ist davon auszugehen, dass die oben beschriebenen Methoden auch weiterhin in vielen Bereichen, von der chemischen Biologie bis zur Wirkstoffforschung, eine breite Anwendung finden werden.[^174^, ^175^, ^176^]

## 9 Synthese von GPI‐modifizierten Peptiden und Proteinen

Die erste Totalsynthese des nativen GPI‐Ankers wurde in den späten 1990ern veröffentlicht.^[177]^ Seit diesem richtungweisenden Bericht wurden einige synthetische Zugänge zu GPI‐Ankern veröffentlicht und an anderer Stelle zusammengefasst.[^178^, ^179^] Während die ersten Synthesen eines GPI‐Ankers erhebliche Leistungen der organischen Synthese darstellten, waren die Moleküle nicht mit passenden Funktionalitäten ausgestattet, um sie mit einem Peptid oder Protein zu verknüpfen. Ein großer Fortschritt im Bereich der lipidierten Proteinsynthese war eine konvergente Strategie, um ein GPI‐verankertes CD52‐Antigen‐Peptid (12 AS) zu erhalten, von Guo und Mitarbeitern im Jahr 2004.^[180]^ Die Gruppe erschloss das CD52‐Glykopeptid und den GPI‐Anker separat und ligierte die beiden Fragmente dann in einer HOBt/EDC‐vermittelten Kupplung. Dieser Arbeit folgten schnell Veröffentlichungen, die die Synthese von GPI‐verankerten Protein‐Analoga beschrieben, beispielsweise eine GFP‐GPI‐^[181]^ und EYFP‐GPI‐Nachahmung.^[182]^ In einer alternativen Strategie haben Guo und Mitarbeiter eine Sortase‐A‐vermittelte Ligation genutzt, um Peptide und kleine Proteine mit GPI‐Ankern zu versehen.^[183]^ Allerdings hatte dieser enzymatische Ansatz zwei Nachteile: 1) Die Anbringung von einem oder zwei nicht‐nativen Gly‐Resten an die Phosphoethanolamingruppe des GPI‐Ankers war notwendig für die Sortase‐A‐Erkennung, und 2) die Erkennungssequenz (LPXTG), die in den C‐Terminus des Proteins eingebracht werden musste, führte zu einer nicht‐nativen Ligationsnarbe im final modifizierten Protein. Nichtsdestotrotz konnte diese Strategie für die effiziente Vorbereitung von Analoga der humanen CD52‐ und CD24‐Antigene sowie eines GPI‐verankerten MUC1 mit einer kurzen Peptidsequenz des Tumor‐assoziierten Proteins genutzt werden.

Ein anderer schlagkräftiger Ansatz, um GPI‐Anker und Peptide zu verknüpfen, nutzt die NCL. Nakahara und Mitarbeiter waren die Ersten, die Thioesterpeptide und Cys‐beinhaltendes GPI über eine NCL‐basierte Methode ligierten,^[184]^ während Bertozzi und Mitarbeiter EPL nutzten, um GPI‐Analoga mit rekombinanten Proteinen zu fusionieren. Sie haben diese Strategie insbesondere genutzt, um GPI‐modifizierte GFP‐Konstrukte zu erschließen, die es ihnen ermöglichten, den Effekt dieser Lipide auf Protein‐Membran‐Assoziation und Membrandiffusion zu untersuchen.^[46]^ Aufbauend auf diesen erfolgversprechenden Studien waren Becker, Seeberger und Mitarbeiter in der Lage, eine robuste semisynthetische Strategie für die Herstellung von homogenem GPI‐verankertem rekombinantem Prion‐Protein (rPrP, **79**), basierend auf einer NCL‐Plattform, zu entwickeln.^[185]^ Hierfür wurde ein synthetischer, Cys‐markierter GPI‐Anker (**80**) mittels NCL an ein exprimiertes rPrP mit einem C‐terminalen MESNa‐Thioester (**81**) ligiert (Schema 9 A). Die Ligation wurde bei pH 7,8 und in Anwesenheit eines Thiophenols als Thioladditiv durchgeführt, was zu einer erfolgreichen Bildung des GPI‐verankerten Proteins führte. Es sollte angemerkt werden, dass die Zugabe von Detergenzien oder Lipiden während der Ligation (die in standardmäßigen 6 M Gdn⋅HCl, 0,3 M NaP_i_‐Puffer durchgeführt wurde) nicht notwendig war und dass der Überschuss an GPI‐Anker nach der Reaktion zurückgewonnen und recycelt werden konnte. Vor kurzen haben Varón Silva und Mitarbeiter diese Technologie durch die Integrierung einer Eintopf‐Ligationsstrategie weiter verbessert, um komplexe GPI‐verankerte Proteine semisynthetisch herzustellen (Schema 9 B).^[186]^ Beispielsweise konnte ein ähnlicher synthetischer GPI‐Anker, der Cys enthält (**82**), mit einem aktiven eGFP‐Proteinthioester ligiert werden, der in situ aus dem entsprechenden Protein‐Npu‐Inteinintermediat (**83**) gebildet wurde, um homogenes GPI‐verankertes eGFP (**84**) zu erhalten. Eine ähnliche Strategie wurde erfolgreich für die Semisynthese von Thy1‐ und *Plasmodium‐berghei‐ANKA*‐MSP119‐Proteinen genutzt, die beide homogene GPI‐Anker aufweisen, wobei hier längere Reaktionszeiten notwendig waren.

**Scheme 9:**
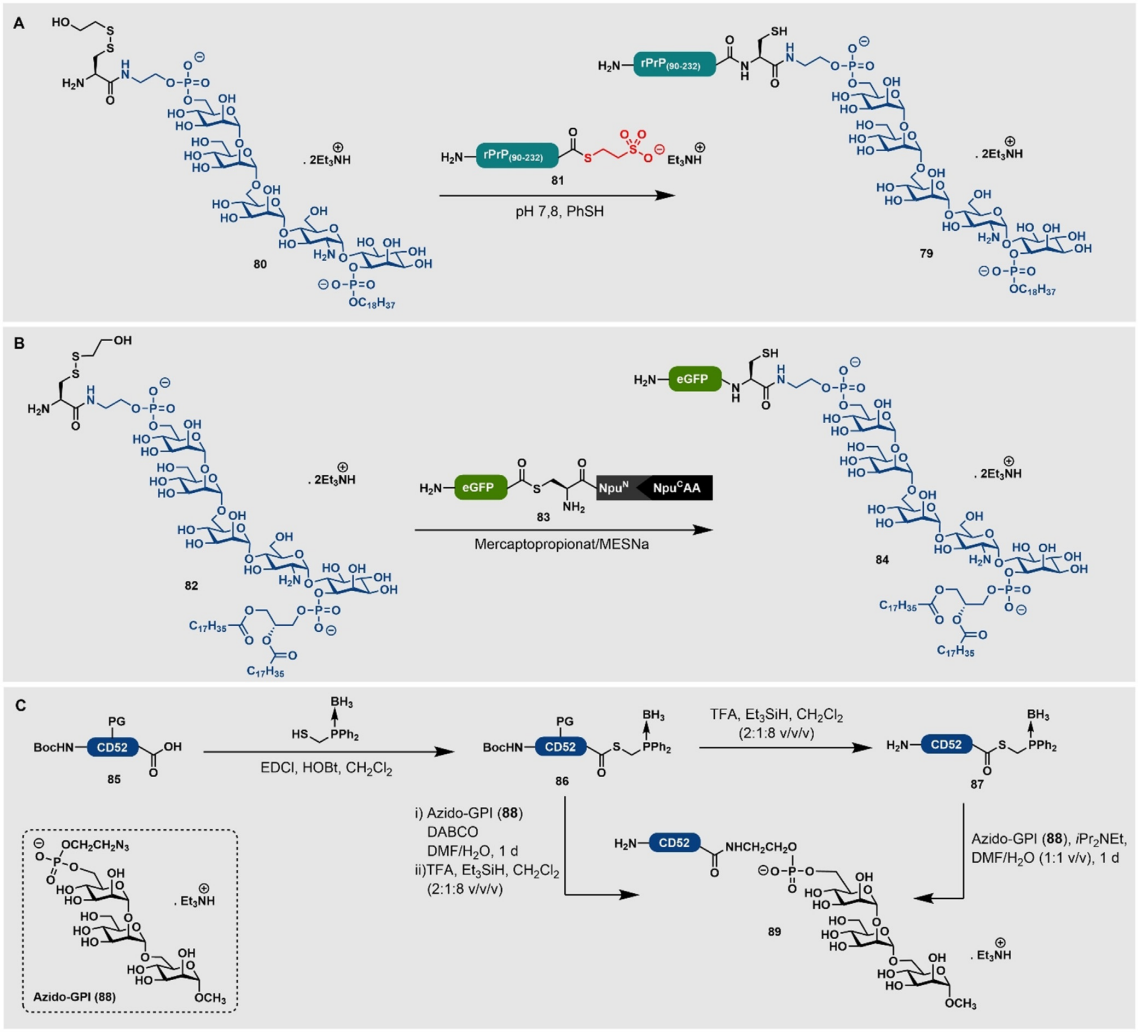
A) NCL‐basierte Zusammenlagerung von einem Cys‐funktionalisierten GPI‐Anker (blau) und einem rekombinanten Prionprotein (rPrP_(90–232)_ mit einem C‐terminalen MESNa‐Thioester (rot)) von Seeberger und Mitarbeitern.^[185]^ B) Eintopf‐NCL eines Cys‐funktionalisierten GPI‐Ankers (blau) mit einem eGFP‐Npu‐Intein‐Intermediat.^[186]^ C) Staudinger‐basierte Synthese des Trimannose‐GPI‐modifizierten CD52 von Guo und Mitarbeitern.^[187]^ Das Peptid wurde mittels Fmoc‐SPPS an einem Säure‐sensitiven 2‐CTC‐Harz synthetisiert. Das Seitenketten‐geschützte CD52 wurde in den entsprechenden Phosphinothioester umgewandelt und anschließend über Staudinger‐Ligation mit einem Azid‐funktionalisierten GPI modifiziert. SG=Schutzgruppe.

Obwohl sowohl Sortase‐A‐ als auch NCL‐basierte Ligationsstrategien effiziente Mittel zur Anknüpfung von synthetischen GPI‐Molekülen an Proteine darstellen, werden in beiden Fällen nicht‐native Protein‐GPI‐Verknüpfungen genutzt, entweder durch eine zusätzliche peptidische Erkennungssequenz (im Fall der Sortase‐Ligation) oder ein Cys‐Rest nach dem NCL‐Schritt. Vor diesem Hintergrund und im Hinblick auf echte native Protein‐GPI‐Konstrukte haben Guo und Mitarbeiter eine Methode für die Kupplung von GPI an Peptide und Proteine mittels spurloser Staudinger‐Ligation entwickelt (Schema 9 C).^[187]^

In einem Beispiel dieser Methode wurde ein CD52‐Peptid (**85**) mittels Fmoc‐Strategie‐SPPS an einem Säure‐sensitiven 2‐CTC‐Harz synthetisiert, und nach der Abspaltung vom Harz wurde es in den entsprechenden Phosphinothioester (**86**) umgewandelt. Dieser konnte durch Säurebehandlung entschützt werden, um den Phosphinothioester **87** zu erhalten. Die Ligation von **86** oder **87** mit einem Azid‐funktionalisierten GPI (**88**) verlief reibungslos und ergab das humane CD52‐Antigen mit einer komplett nativen Verknüpfung zwischen dem Protein und dem GPI‐Anker (**89**). Diese strategische Nutzung der spurlosen Staudinger‐Ligation schafft die Voraussetzung für die Herstellung vieler weiterer nativer, GPI‐verankerter Proteine in der Zukunft, wie beispielsweise das CD48‐Antigen und die Carboanhydrase IV (beide durch ein C‐terminales Ser‐GPI‐verankert) oder den Eph‐Rezeptor‐Liganden, Ephrin A5 (durch ein C‐terminales Asn‐GPI‐modifiziert). Diese Proteine wurden bis heute noch nicht in ihrer homogenen Form untersucht. Von zentraler Bedeutung für diese Studien werden die Implementierung kürzlich entwickelter Vorhersagemethoden zur Identifizierung neuer GPI‐verankerter Proteine, wie beispielsweise PredGPI,^[188]^ und die Ausweitung der Phosphinothioesterbildung auf größere und rekombinante Proteine, für die sich Intein‐basierte Methoden möglicherweise als eine grundlegende Technologie erweisen könnten. Allerdings kann die spurlose Staudinger‐Ligation im Vergleich zu anderen Ligationsmethoden unter niedrigen Reaktionsgeschwindigkeiten leiden, sodass es möglich ist, dass solche größeren Protein‐Phosphinothioester nicht so effizient ligieren wie die kleineren Peptidbeispiele, die bis heute getestet wurden.^[189]^

Ein limitierender Faktor in den oben beschriebenen Ansätzen ist die Verfügbarkeit von ausreichenden Mengen des funktionalisierten GPI‐Ankers, da diese mittels mehrstufiger Syntheserouten schwierig zu synthetisieren sind. Darüber hinaus wurden Schwierigkeiten bei der Handhabung dieser nativ lipidmodifizierten Proteine aufgrund von Löslichkeitsproblemen und/oder amphipathischen Eigenschaften festgestellt, was dazu führte, dass viele Wissenschaftler sich in synthetischen und semisynthetischen Ansätzen der Nutzung von weniger komplexen GPI‐Kernstrukturen zugewandt haben.^[190]^ Eine mögliche Lösung ist die Nutzung von natürlichen GPI‐Ankern, die von Zellen produziert wurden, allerdings gibt es bis jetzt erst wenige Beispiele hierfür. Schumacher et al. beschreiben beispielsweise die Generierung eines GPI‐verankerten Peptides mit einem freien, N‐terminalen Cys in Hefe, das in Ligationsreaktionen mit Peptid‐ oder Proteinthioestern genutzt werden kann.^[191]^ Dhar und Mootz hingegen haben die innovative Nutzung eines Spli‐Intein‐basierten (*Npu* DnaE) Systems veröffentlicht, das auf exprimierten GPI‐verankerten Peptiden basiert, die mit einem C‐terminalen Inteinsegment fusioniert sind, mit anschließendem Transspleißen mit einem anderen Protein, das ein N‐terminales Intein trägt (in diesem Fall das Modell‐Protein eGFP).^[192]^

## 10 Zusammenfassung und Ausblick

Die in diesem Aufsatz diskutierten Methoden und Beispiele beschreiben den aktuellen Status der (semi)synthetischen Strategien, um lipidierte Peptide und Proteine zu generieren. Um einen leicht zugänglichen Überblick über diesen Bereich zu geben, haben wir die wichtigsten Lipidklassen in Peptiden und Proteinen kurz und bündig dargestellt. Es wird eine kurze Beschreibung der wichtigsten chemischen Ansätze zur Herstellung lipidierter Peptide und Proteine gegeben. Wir haben die Vorteile und Herausforderungen der einzelnen Strategien hervorgehoben, die wir bei der Darstellung spezifischer Beispiele für jede Klasse der Lipidierung näher erläutern. Diese Beispiele verdeutlichen, dass unsere Fähigkeit, homogen lipidmodifizierte Proteine aus Segmenten zusammenzufügen, die durch SPPS und rekombinante Expression hergestellt wurden, im letzten Jahrzehnt erheblich verbessert wurde und als Grundlage für eine Reihe wichtiger Entdeckungen in Biologie und Medizin diente.

Die empfindliche Natur der Bindungen zwischen Lipiden und Proteinen (z. B. Thioester), die chemische Komplexität spezifischer Lipidmodifikationen (z. B. GPI‐ oder PE‐Anker) sowie die Auswirkungen von Lipidmodifikationen auf die Ligationsausbeuten, aufgrund erhöhter Hydrophobie oder Amphipathie, machen diese synthetischen und semisynthetischen Bemühungen jedoch immer noch zu einer großen Herausforderung. Obwohl diese Einschränkungen in manchen Fällen umgangen werden können, indem nicht‐native Verknüpfungen zwischen Lipid(en) und Proteinen eingebracht werden, gibt es Anlass zur Besorgnis über die funktionellen Konsequenzen der Einführung dieser künstlichen Variationen.

Trotz signifikanter Fortschritte im letzten Jahrzehnt bleibt unser Wissen bezüglich der funktionellen Rollen von verschiedenen Lipidmodifikationen nach wie vor unvollständig. Es ist jedoch davon auszugehen, dass Erweiterungen und Verbesserungen der ligationsbasierten Proteinsynthesemethoden wie NCL, EPL, DSL, STL und KAHA das Feld weiter voranbringen werden. Ein wichtiger Aspekt wird die Durchführung von Ligationsreaktionen bei niedrigen Konzentrationen sein. Dies kann sowohl durch Erweiterungen der DSL‐Methode als auch durch den Einsatz von Löslichkeits‐Tags an lipidierten Peptidsegmenten erreicht werden, um NCL‐, EPL‐ und sogar Protein‐Transspleiß (PTS)‐Reaktionen zu verbessern, die auf Split‐Inteinen beruhen.[^2^, ^77^]

Wir gehen davon aus, dass zusätzlicher Fortschritt durch eine Kombination aus den hochentwickelten Ligationsstrategien, die oben beschrieben wurden, und der Entwicklung von neuen chemo‐ und regioselektiven Modifikationsreaktionen (z. B. Lipidierung von ungeschützten Cys) generiert wird.[^193^, ^194^] In diesem Bereich wurde in letzter Zeit eine Reihe neuer Verfahren entwickelt, besonders angetrieben durch den Bedarf an effizienten Konjugationsreaktionen zur Herstellung selektiv modifizierter Proteintherapeutika. Diese Methoden können jetzt für die Modifikation von Volllängenproteinen genutzt werden, auch um Handhabungsprobleme bei der Proteinsynthese zu vermeiden. In ähnlicher Weise bieten enzymvermittelte Strategien zwei verschiedene Möglichkeiten für die Synthese von lipidierten Proteinen. Erstens für den Proteinaufbau, durch die Nutzung von enzymvermittelten Ligationsstrategien (z. B. durch Nutzung von gentechnisch hergestellten Proteasen oder spezifischen Peptid‐Ligasen)^[195]^ und zweitens für die enzymatische Lipidierung von Proteinen.^[196]^ Die Anwendung dieser Methoden wird einen effizienteren und robusteren Zugang zu lipidierten Proteinen ermöglichen und damit die Untersuchung der Lipidierung in grundlegenden biologischen Studien beschleunigen, aber auch lipidierte Proteine für den Biotechnologie‐ und Pharmasektor bereitstellen.

## Interessenkonflikt

Die Autoren erklären, dass keine Interessenkonflikte vorliegen.

## Biographical Information

*Cameron C. Hanna erhielt 2015 seinen BSc(Hons) mit Schwerpunkt Chemie an der University of Otago, Neuseeland. Er promovierte 2020 an der University of Sydney, Australien, unter der Anleitung von Prof. Richard Payne. Nach einem Postdoktorat bei Prof. Dame Margaret Brimble ist er derzeit Postdoktorand im Labor von Prof. Bradley Pentelute am MIT, wo sich seine Forschung auf die Entwicklung von Peptid‐Wirkstoffentdeckungs‐Plattformen auf der Grundlage von Phagen‐Display sowie auf neue Methoden zur selektiven Veränderung von Proteinen in lebenden Zellen konzentriert*.

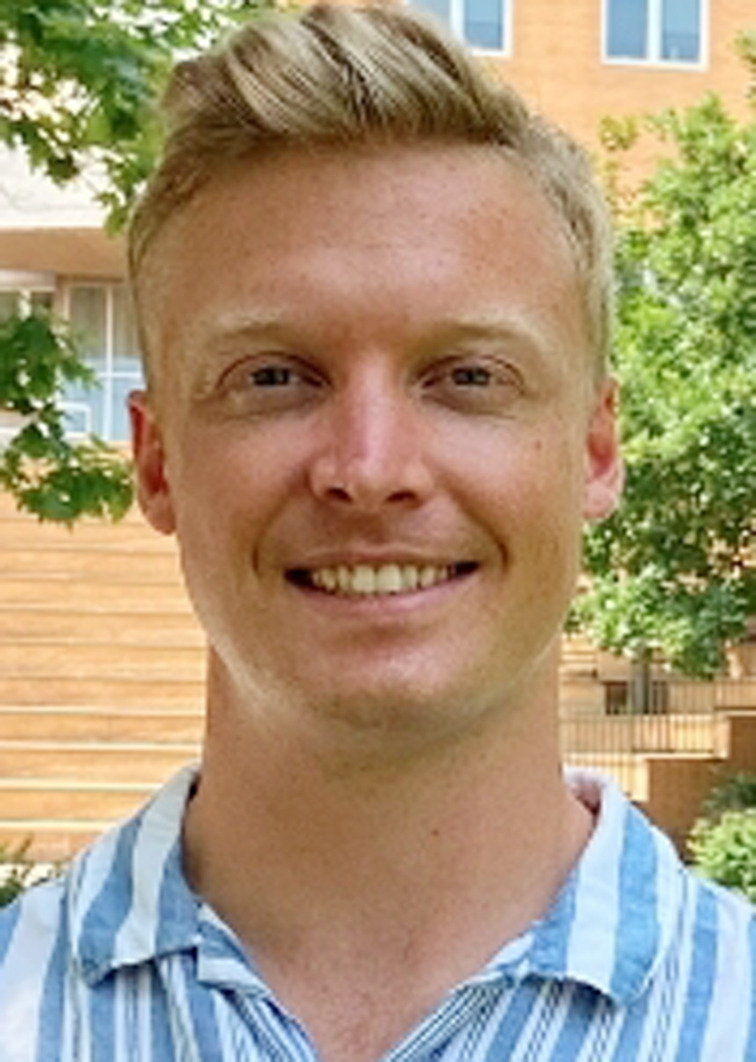

## Biographical Information

*Julia Kriegesmann erhielt 2016 ihren MSc in Chemischer Biologie von der TU Dortmund, Deutschland, und ihren Doktortitel von der Universität Wien, Österreich, unter der Betreuung von Prof. Christian F. W. Becker im Jahr 2021. Sie arbeitet derzeit als Postdoktorandin in der Becker‐Arbeitsgruppe, in der sie ihre Arbeit an der selektiven Modifikation von Cysteinen in Peptiden und Proteinen fortsetzt*.

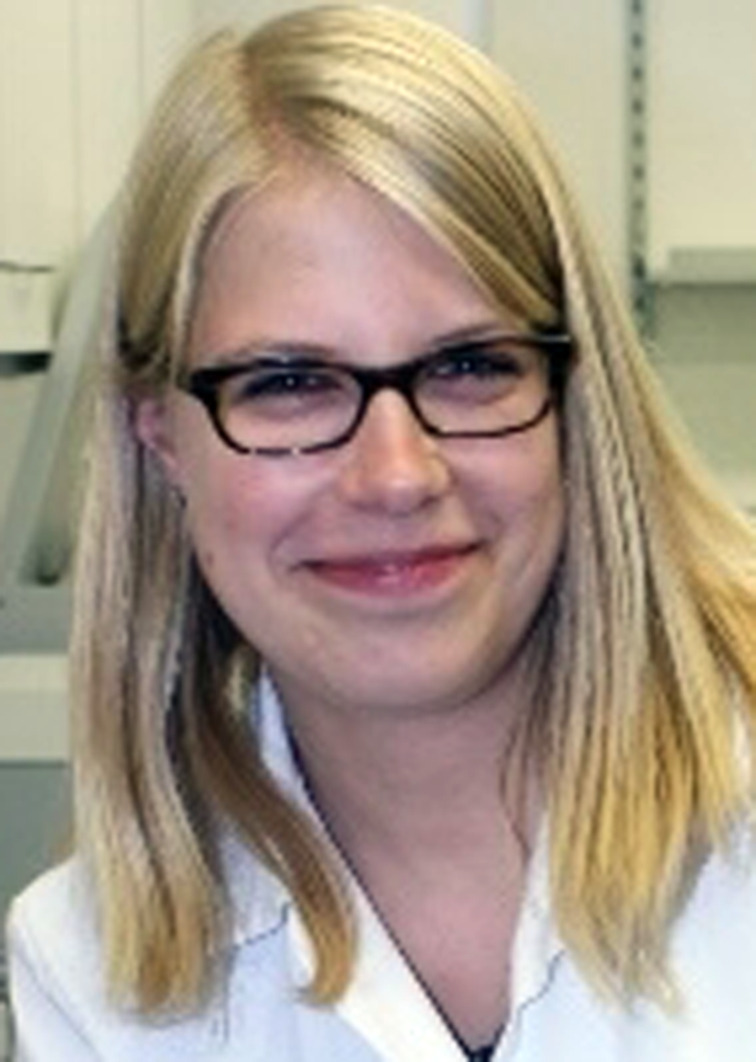

## Biographical Information

*Luke J. Dowman schloss 2015 sein Studium der Chemie und Biochemie an der University of Sydney, Australien, mit einem BSc (Advanced) (Hons) ab und promovierte 2020 an der University of Sydney bei Prof. Richard Payne. Während dieser Zeit befasste er sich unter anderem mit der Entwicklung eines Reaktionssystems für die schnelle und selektive Funktionalisierung von Peptiden und Proteinen. Er ist derzeit Postdoktorand in der Payne‐Gruppe und am ARC Centre of Excellence for Innovations in Peptide and Protein Science, wo er neue Funktionalisierungsverfahren von Proteinen erforscht*.

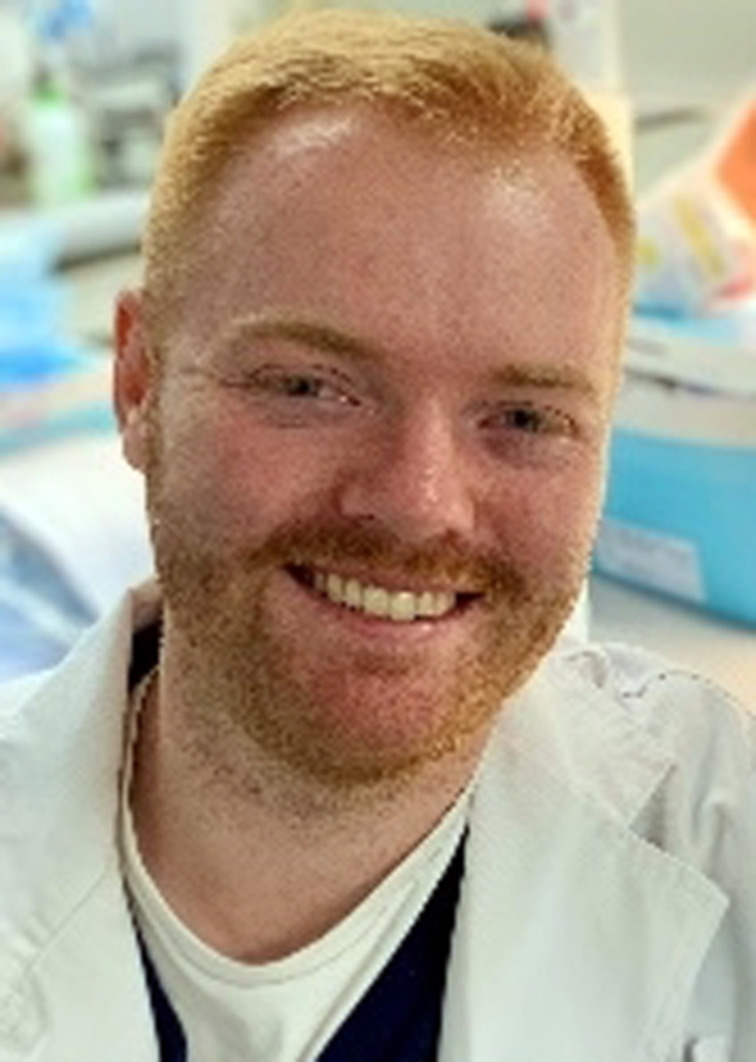

## Biographical Information

*Christian F. W. Becker studierte Chemie an der Universität Dortmund (Diplom 1998). Nach dem Doktorat 2001 an der gleichen Universität arbeitete er 2002–2003 als Postdoktorand bei Gryphon Therapeutics. Er wurde 2004 Gruppenleiter am MPI in Dortmund und 2007 Professor für Proteinchemie an der TU München. 2011 wurde er Professor und Leiter des Institutes für Biologische Chemie an der Universität Wien und 2020 Gründungsvorstand der Vienna Doctoral School in Chemistry (DoSChem). Er entwickelt (bio)chemische Methoden, um Peptide und Proteine mit sonst unzugänglichen (posttranslationalen) Modifikationen zu generieren*.

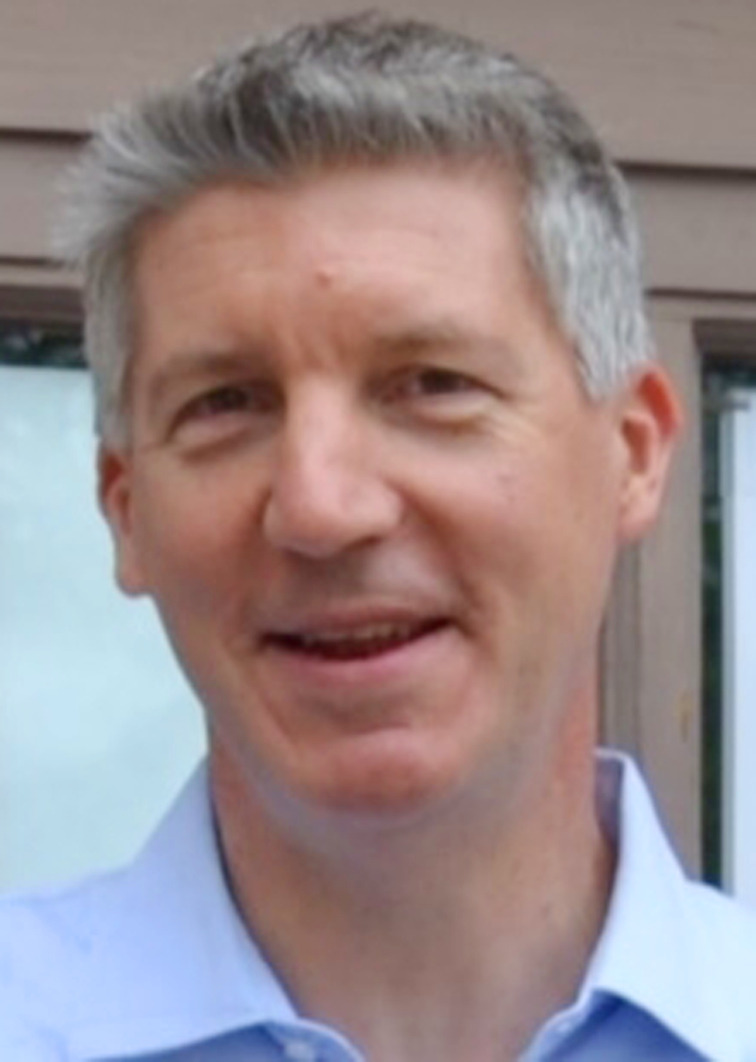

## Biographical Information

*Richard J. Payne studierte Chemie an der University of Canterbury, Neuseeland, (BSc(Hons)) und promovierte 2006 an der University of Cambridge bei Prof. Chris Abell FRS FMedSci. Nach einem Postdoktorat am Scripps Research Institute bei Prof. Chi‐Huey Wong (im Rahmen eines Lindemann‐Stipendiums) begann er 2008 seine eigenständige Karriere an der University of Sydney. Seit 2015 ist er Professor für Organische Synthese und Chemische Biologie und derzeit stellvertretender Direktor des ARC Centre of Excellence for Innovations in Peptide and Protein Science. Er entwickelt Methoden für den Zugang zu komplexen Biomolekülen, um wichtige Probleme in Biologie und Medizin zu lösen*.